# Dual functions for the ssDNA-binding protein RPA in meiotic recombination

**DOI:** 10.1371/journal.pgen.1007952

**Published:** 2019-02-04

**Authors:** Baolu Shi, Jiangyang Xue, Hao Yin, Rui Guo, Mengcheng Luo, Lan Ye, Qinghua Shi, Xiaoyan Huang, Mingxi Liu, Jiahao Sha, P. Jeremy Wang

**Affiliations:** 1 State Key Laboratory of Reproductive Medicine, Nanjing Medical University, Nanjing, China; 2 Department of Biomedical Sciences, University of Pennsylvania School of Veterinary Medicine, Philadelphia, Pennsylvania, United States of America; 3 Center for Reproduction and Genetics, Nanjing Medical University Affiliated Suzhou Hospital, Suzhou, Jiangsu, China; 4 USTC-SJH Joint Center for Human Reproduction and Genetics, School of Life Sciences, University of Science and Technology of China, Hefei, China; 5 Department of Tissue and Embryology, Hubei Provincial Key Laboratory of Developmentally Originated Disease, School of Basic Medical Sciences, Wuhan University, Wuhan, China; Cornell University, UNITED STATES

## Abstract

Meiotic recombination permits exchange of genetic material between homologous chromosomes. The replication protein A (RPA) complex, the predominant ssDNA-binding complex, is required for nearly all aspects of DNA metabolism, but its role in mammalian meiotic recombination remains unknown due to the embryonic lethality of RPA mutant mice. RPA is a heterotrimer of RPA1, RPA2, and RPA3. We find that loss of RPA1, the largest subunit, leads to disappearance of RPA2 and RPA3, resulting in the absence of the RPA complex. Using an inducible germline-specific inactivation strategy, we find that loss of RPA completely abrogates loading of RAD51/DMC1 recombinases to programmed meiotic DNA double strand breaks, thus blocking strand invasion required for chromosome pairing and synapsis. Surprisingly, loading of MEIOB, SPATA22, and ATR to DNA double strand breaks is RPA-independent and does not promote RAD51/DMC1 recruitment in the absence of RPA. Finally, inactivation of RPA reduces crossover formation. Our results demonstrate that RPA plays two distinct roles in meiotic recombination: an essential role in recombinase recruitment at early stages and an important role in promoting crossover formation at later stages.

## Author summary

Meiosis, a process unique to germ cells, results in production of haploid gametes. Meiotic recombination, a hallmark of meiosis, together with random segregation of homologous chromosomes, generates genetic diversity in haploid gametes at every generation so that each gamete has a unique genetic composition. Such genetic diversity in gametes is important for evolution. Here we report the functional requirement of RPA in meiotic recombination in mouse. RPA is a ubiquitously expressed ssDNA-binding complex and is essential for DNA replication. Mutations in RPA cause lethality. Using an inducible Cre-mediated deletion approach, we find that RPA is required for meiotic recombination in mouse. Inactivation of RPA causes absence of DNA recombinases RAD51 and DMC1 at DNA double-strand breaks, resulting in a block in meiotic recombination at the zygotene stage. In contrast, the ssDNA-binding MEIOB/SPATA22 heterodimers and ATR still form foci on meiotic chromosomes in the absence of RPA. Moreover, inactivation of RPA reduces crossover formation in pachytene spermatocytes. In conclusion, RPA plays two stage-specific functions in the early recombinase recruitment and the late crossover formation respectively during meiotic recombination.

## Introduction

During sexual reproduction, meiotic recombination permits reciprocal exchange of genetic materials between homologous chromosomes and ensures faithful chromosome segregation [1, 2]. Abnormalities in meiotic recombination are a leading cause of aneuploidy, infertility, and pregnancy loss in humans [3]. Meiotic recombination is initiated by the formation of programmed DNA double strand breaks (DSBs) in germ cells and involves a large number of single-stranded DNA (ssDNA)-binding proteins [2]. These DSBs undergo end resection to generate 3’ ssDNA overhangs; subsequent loading of RAD51 and DMC1 recombinases and other proteins enables strand invasion into duplex DNA for homologue pairing and recombination intermediate formation [4–7]. All meiotic DSBs are repaired but only a subset lead to crossovers, which are critical for proper segregation of homologous chromosomes during the first meiotic cell division.

The replication protein A (RPA) complex, comprised of RPA1, RPA2, and RPA3, is the predominant ssDNA-binding heterotrimeric complex in DNA metabolism [8]. RPA1, the largest subunit, is responsible for the majority of ssDNA-binding activity of the RPA complex. RPA protects ssDNA from degradation and prevents secondary structure formation. RPA interacts with both RAD51 and DMC1 [9], suggesting that RPA may direct RAD51 and DMC1 to these ssDNA overhangs. RAD51 and DMC1 form nuclear complexes on meiotic chromosomes [10, 11]. The Hop2-Mnd1 heterodimer interacts with the RAD51/DMC1 recombinases and stimulates their enzymatic activity [12]. MEIOB is a meiosis-specific ssDNA-binding RPA1 homologue [13, 14]. The MEIOB/SPATA22 heterodimer interacts with RPA and could also position critical proteins for meiotic recombination [15]. These interactions suggest that RPA may play multiple roles in meiotic recombination [16]. A long-standing question remains as to whether RPA is required for loading of these ssDNA-binding proteins to DSBs *in vivo*.

RPA is ubiquitously expressed and essential for DNA replication, repair, and recombination [8]. However, the physiological role of RPA in mammalian meiosis remains elusive, due to embryonic lethality of the *Rpa1* mutation in mice [17]. As a result, the interplay of RPA with other ssDNA-binding proteins during meiotic recombination *in vivo* remains enigmatic. Here we employed a germ cell-specific inducible deletion approach and uncovered the functions of RPA in meiosis.

## Results

### The stability of the RPA heterotrimeric complex depends on RPA1

**Fig 1:**
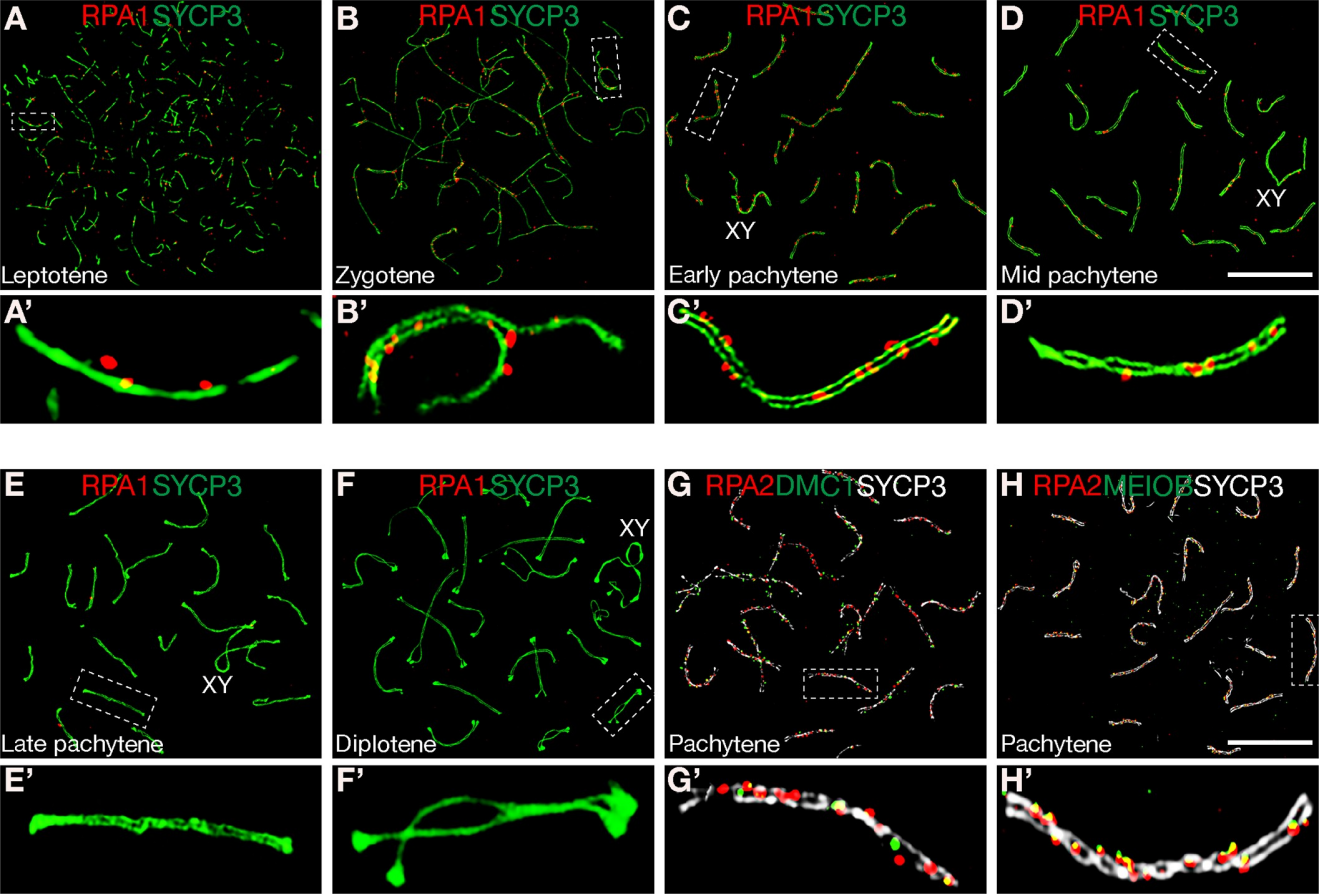
Super-resolution localization of RPA, DMC1 and MEIOB on meiotic chromosomes. Spermatocytes from adult testes were subjected to immunofluorescent analysis with various antibodies. (**A-F**) Localization of RPA1 in leptotene through diplotene spermatocytes. Early, mid, and late pachytene stages were distinguished based on the XY morphology, SYCP3 staining intensity and the accumulation of SYCP3 at the SC ends as previously described [41]. (**G**) Double immunolocalization analysis of RPA2 and DMC1 in early pachytene spermatocytes. (**H**) Co-localization of RPA2 and MEIOB in early pachytene spermatocytes. Enlarged view of boxed individual chromosomes (**A-H**) is shown in the bottom panels (**A′-H′**) respectively. Scale bars, 10 μm.

Using super-resolution imaging microscopy (SIM), we examined the localization pattern of RPA and other meiotic proteins in mouse spermatocytes. RPA is a heterotrimer of RPA1, RPA2, and RPA3 [8]. These three RPA subunits are expected to colocalize in the same foci. Indeed, by conventional immunofluorescence microscopy, we previously showed that RPA1 always colocalized with MEIOB and that RPA2 also always colocalized with MEIOB [13]. By SIM, we found that RPA1 foci were present in leptotene, zygotene, early pachytene, and mid pachytene spermatocytes, but absent in late pachytene and diplotene spermatocytes (Fig 1A–1F). We next examined colocalization of RPA2 with other DNA-binding proteins that are known to be involved in meiosis. While most RPA2 foci did not overlap with DMC1 foci (Fig 1G), RPA2 foci always colocalized with MEIOB foci (Fig 1H). These results were consistent with previous conventional immunolocalization findings, suggesting that RPA may function in meiotic recombination in mouse [13, 18].

To overcome the expected embryonic lethality of *Rpa1* inactivation, we generated an *Rpa1* floxed (*Rpa1*^fl^) allele that allows for germ cell-specific inactivation of *Rpa1*. We first produced *Rpa1*^fl/-^ *Ddx4*-Cre males. *Ddx4*-Cre begins to express only in germ cells at embryonic day 15 [19]. *Rpa1*^fl/-^ *Ddx4*-Cre males were viable but sterile. Testes from 10-week-old *Rpa1*^fl/-^ *Ddx4*-Cre males lacked all germ cells (S1A Fig). We also generated *Rpa1*^fl/-^ *Stra8*-Cre males. *Stra8*-Cre begins to express in spermatogonia postnatally, prior to meiosis [20]. Testes from adult *Rpa1*^fl/-^ *Stra8*-Cre males displayed a heterogeneous phenotype: some tubules were nearly depleted of all germ cells, while other tubules had apparently normal spermatogenesis (S1B Fig), presumably due to inefficient *Stra8*-Cre-mediated deletion as observed previously [21]. Although these genetic studies revealed an essential role for RPA1 in spermatogenesis, the loss of all germ cells in testes from *Rpa1*^fl/-^ *Ddx4*-Cre males or the presence of all germ cells in testes from *Rpa1*^fl/-^ *Stra8*-Cre males precluded investigating its function in meiosis.

**Fig 2:**
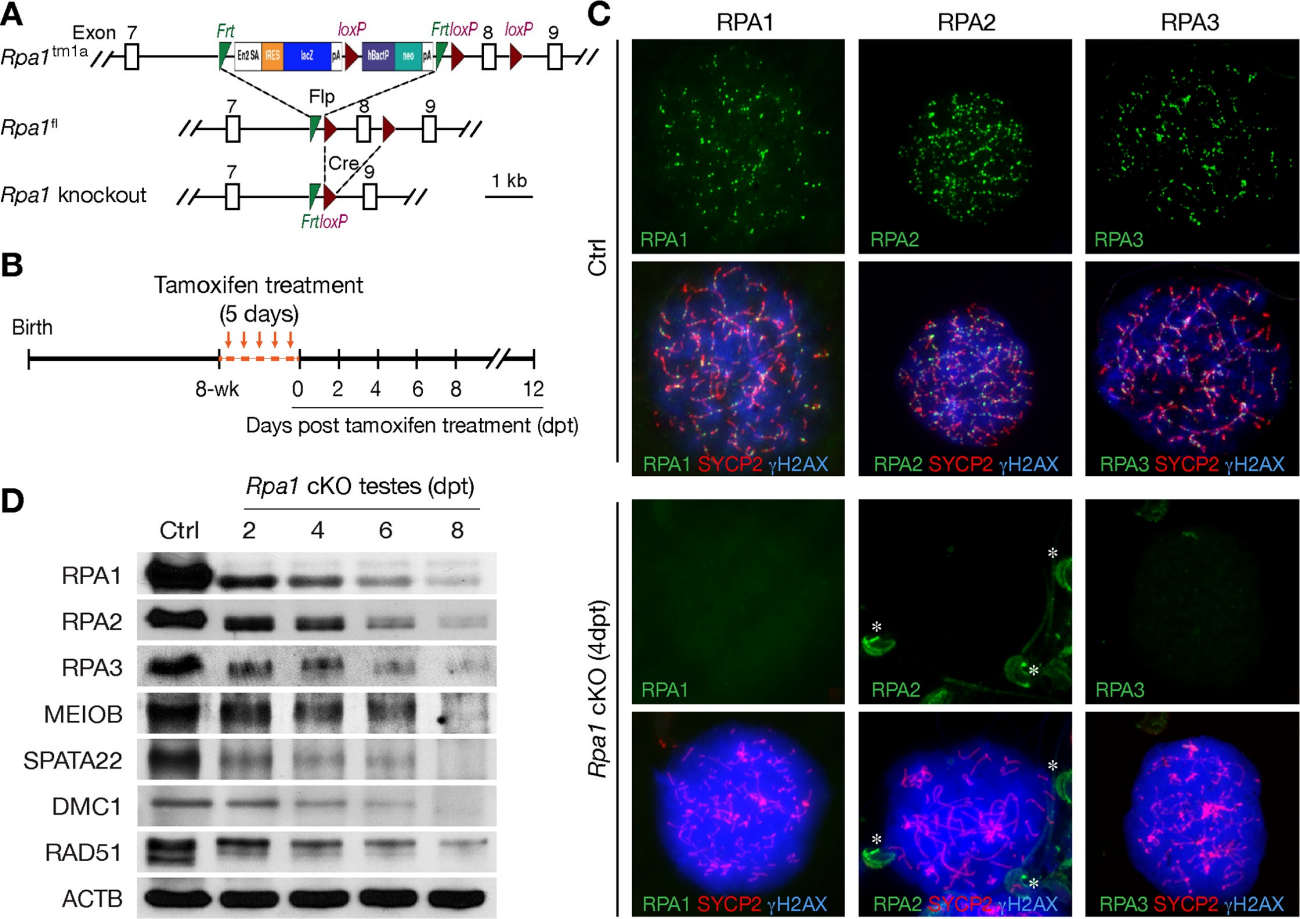
Tamoxifen-inducible Cre-mediated *Rpa1* deletion strategy during male meiosis. (A) Schematic diagram of various *Rpa1* alleles: *Rpa1*^tm1a^, *Rpa1*^fl^ (floxed allele), and *Rpa1* knockout. Cre-mediated deletion of exon 8 (encoding residues 182–226) is expected to cause a frameshift in the resulting mutant transcript. FLP, FLP1 recombinase; Cre, Cre recombinase. Frt and loxP sites are shown. (B) Tamoxifen treatment regimen. (C) Absence of RPA1, RPA2, and RPA3 foci in *Rpa1*^cKO^ zygotene-like spermatocytes. RPA1, RPA2, and RPA3 form many foci on synaptonemal complexes in control leptotene spermatocytes but none in zygotene-like spermatocytes from *Rpa1*^cKO^ testes at 4 days post-tamoxifen treatment. Asterisks indicate the non-specific immunofluorescence signals (RPA2) on sperm heads. (D) Western blot analysis of various ssDNA-binding proteins (RPA1, RPA2, RPA3, MEIOB, SPATA22, DMC1, RAD51) in testes from 8-week control and *Rpa1*^cKO^ mice at 2, 4, 6, and 8 days post-tamoxifen treatment (dpt). ACTB serves as a loading control.

To circumvent these hurdles, we performed tamoxifen-inducible inactivation of *Rpa1* following crossing with *Ddx4*-Cre^ERT2^ mice (Fig 2A) [22]. Intraperitoneal injection of 8-week-old *Rpa1*^fl/fl^ *Ddx4*-Cre^ERT2^ (referred to as *Rpa1*^cKO^) male mice with tamoxifen resulted in the deletion of exon 8, which led to a frame shift in the resulting mutant transcript (Fig 2A and 2B). The abundance of the *Rpa1* transcript (both pre and post exon 8 regions) decreased at days 2, 4, and 6 post tamoxifen treatment, suggesting that the *Rpa1* transcript is depleted due to nonsense mediated decay and/or depletion of spermatocytes (S2 Fig). Immunofluorescence analysis of spread nuclei revealed the absence of RPA1 foci in mutant spermatocytes at 4 days post-tamoxifen treatment (dpt), showing efficient depletion of *Rpa1* in zygotene-like spermatocytes under this regimen (Fig 2C). Interestingly, although RPA2 and RPA3 formed foci in wild type spermatocytes, they failed to form foci on meiotic chromosomes in zygotene-like *Rpa1* mutant spermatocytes (Fig 2C). These results were confirmed by the dramatic reduction in the abundance of RPA1, RPA2, and RPA3 proteins in tamoxifen-treated testes (Fig 2D). These data demonstrate that the stability of both RPA2 and RPA3 depends on RPA1 and that loss of RPA1 causes the depletion of the RPA complex.

### RPA1 is essential for meiotic progression

**Fig 3:**
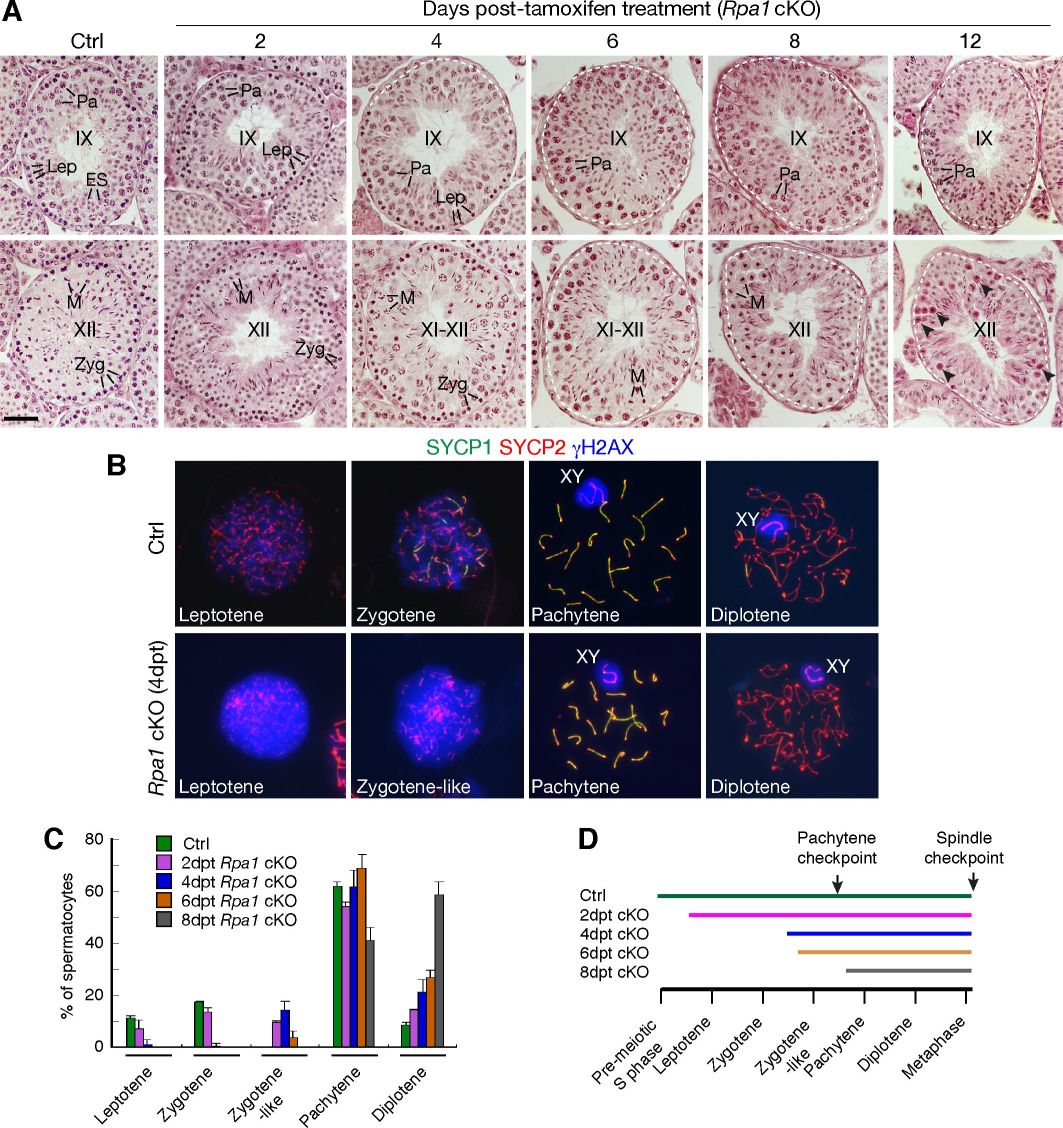
RPA1 is essential for meiosis in males. (A) Histological analysis of testes from 8-week-old control and tamoxifen-treated *Rpa1*^fl/fl^ *Ddx4-*Cre^ERT2^ mice. Cross-sections of Stage IX and XII seminiferous tubules are shown. Dash lines indicate missing germ cells. Arrowheads designate the apparently apoptotic spermatocytes (day 12, Stage XII). Lep, leptotene; Zyg, zygotene; Pa, pachytene; M, metaphase spermatocytes; ES, elongating spermatids. Scale bar, 50 μm. (B) Surface spread analysis of spermatocytes from testes at 4 days post-TMX treatment (dpt). (C) Composition of spermatocytes. Three males per timepoint were analysed. ~200 spermatocytes from each mouse were counted and categorized into different spermatocyte types. The percentage (average ± SD) was plotted. (D) Time-dependent progressive loss of spermatocytes in tamoxifen-treated *Rpa1*^fl/fl^ *Ddx4-*Cre^ERT2^ males. Lines indicate the presence of spermatocytes. Two cell cycle checkpoints (pachytene checkpoint at stage IV and spindle assembly checkpoint at stage XII) are shown. Note that zygotene-like is separated from zygotene for illustration purpose and is not a separate stage of meiosis.

Inactivation of *Rpa1* results in severe defects in meiotic progression in males (Fig 3A). At 4 dpt, *Rpa1*^cKO^ seminiferous tubules (Stage IX) were partially devoid of leptotene spermatocytes (Fig 3A). At 6 dpt, both leptotene (stage IX) and zygotene (stage XII) spermatocytes were lost in *Rpa1*^cKO^ testes. At 8 dpt, in addition to the loss of leptotene and zygotene spermatocytes, there was a partial loss of pachytene spermatocytes (Fig 3A and 3C). At 12 dpt, pachytene spermatocytes exhibited a significant loss (Fig 3A). Progressive loss of spermatocytes was expected to contribute partially to the reduction in protein abundance of RPA and other proteins in the mutant testes (Fig 2D).

We monitored meiotic progression and determined the spermatocyte composition in *Rpa1*^cKO^ males by nuclear spread analysis (Fig 3B and 3C). At 4 dpt, *Rpa1*^cKO^ testes lacked normal zygotene spermatocytes, characterized by partial chromosomal synapsis, but contained defective zygotene-like spermatocytes, which were characterized by lack of synapsis and presence of unusually strong γH2AX (Fig 3B). The γH2AX intensity in both *Rpa1*-deficient leptotene and zygotene-like spermatocytes was significantly higher than wild type, suggesting that DSBs were generated but not repaired in the absence of RPA1 (Fig 3B). Zygotene-like spermatocytes were only present in *Rpa1*^cKO^ testes at 2 to 6 days post tamoxifen treatment with a peak at 2 dpt and 4 dpt (Fig 3C and 3D). Consistent with histological analysis (Fig 3A), only pachytene and diplotene spermatocytes were present in *Rpa1* mutant testes at 8 dpt (Fig 3C and 3D). Apoptosis in spermatocytes increased dramatically in stage IV tubules from *Rpa1*^cKO^ testes (S3 Fig), suggesting that *Rpa1*-deficient zygotene-like spermatocytes were eliminated by the pachytene checkpoint in response to early meiotic defects (Fig 3D).

### RPA1 is required for pre-meiotic S phase DNA replication

**Fig 4:**
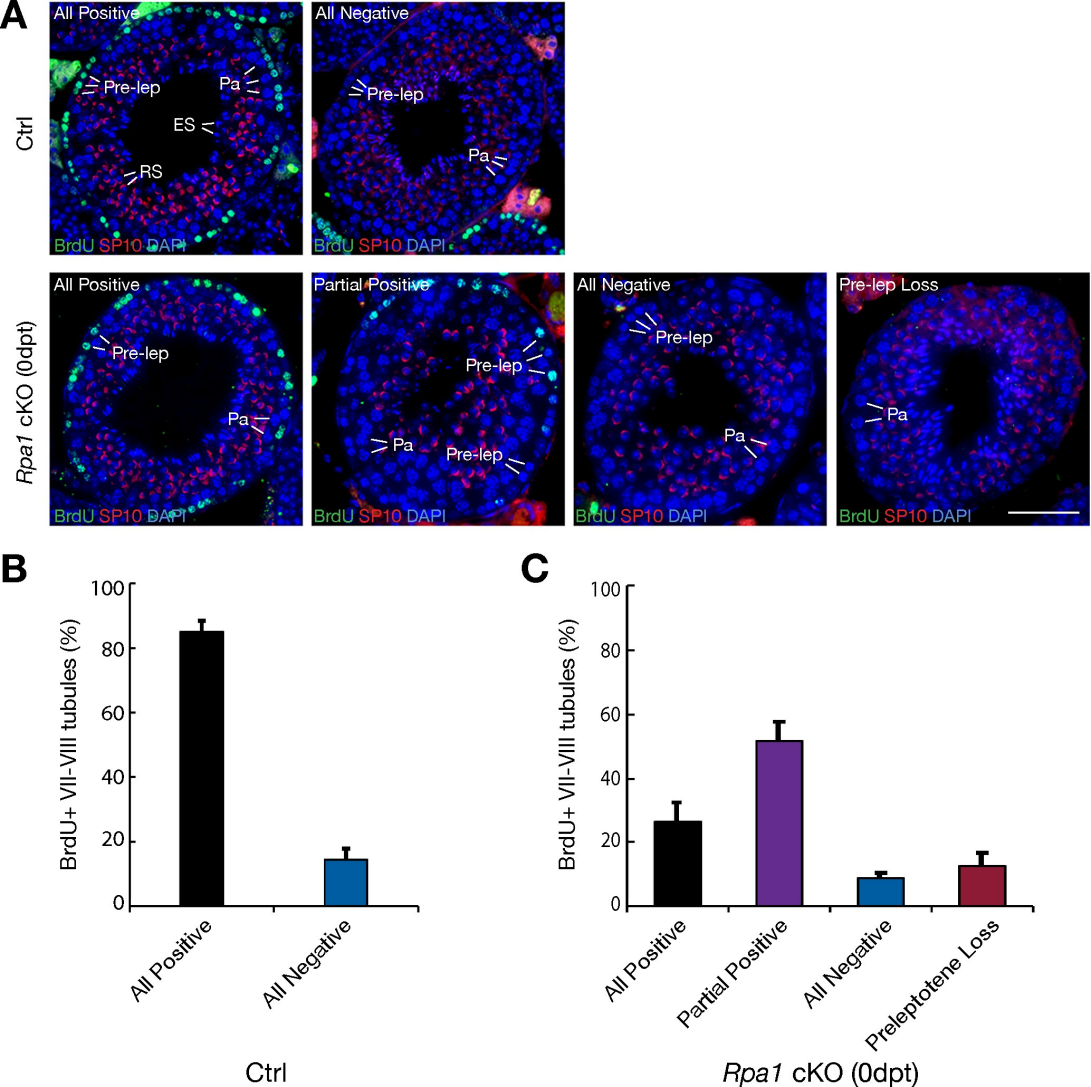
RPA1 is required for pre-meiotic S-phase DNA replication in preleptotene spermatocytes. Pulse labelling of DNA replicating cells was performed with intraperitoneal injection of BrdU. (A) Immunofluorescence analysis of BrdU incorporation in stage VII-VIII seminiferous tubules. The acrosome morphology shown by SP10 immunofluorescence was used for seminiferous tubule staging. All positive tubule: all preleptotene spermatocytes are BrdU-positive; All negative tubule: all preleptotene spermatocytes are BrdU-negative; Partial positive tubule: a fraction of preleptotene spermatocytes are BrdU-positive; Pre-lep loss tubule: absence of preleptotene spermatocytes in stage VII-VIII seminiferous tubules. (B) Percentage of two types of stage VII-VIII tubules in control mice. (C) Percentage of four types of stage VII-VIII tubules from *Rpa1*^cKO^ at 0 day post-tamoxifen treatment. (B, C) Three pairs of control and *Rpa1*^cKO^ males (0 day post tamoxifen treatment) were analyzed. At least thirty five stage VII-VIII tubules were counted for each mouse.

The synchronized depletion of meiotic germ cells in mutant testes suggests that RPA1 is required for pre-meiotic S phase DNA replication in preleptotene spermatocytes. To test this, we performed a BrdU incorporation (pulse-labelling) assay after tamoxifen treatment (0 dpt) (Fig 4A). Preleptotene spermatocytes were only present in stage VII-VIII tubules and were nearly all BrdU-positive in ~80% of such tubules in wild type testes, whereas preleptotene spermatocytes in the remaining 20% of wild type stage VII-VIII tubules were all BrdU-negative (Fig 4B). Partial positive tubules with a mixture of BrdU-positive and BrdU-negative preleptotene spermatocytes were not observed in wild type testes but accounted for 50% of tubules in *Rpa1*^cKO^ testes (Fig 4A and 4C), supporting the requirement for RPA1 in pre-meiotic S phase DNA replication in BrdU-negative preleptotene spermatocytes. While not observed in wild type testes, nearly 10% of stage VII-VIII *Rpa1*^cKO^ tubules lacked preleptotene spermatocytes, suggesting that RPA1 is also essential for mitotic DNA replication in spermatogonia, direct precursors of preleptotene spermatocytes.

### Loading of recombinases RAD51 and DMC1 to DSBs is dependent on RPA

**Fig 5:**
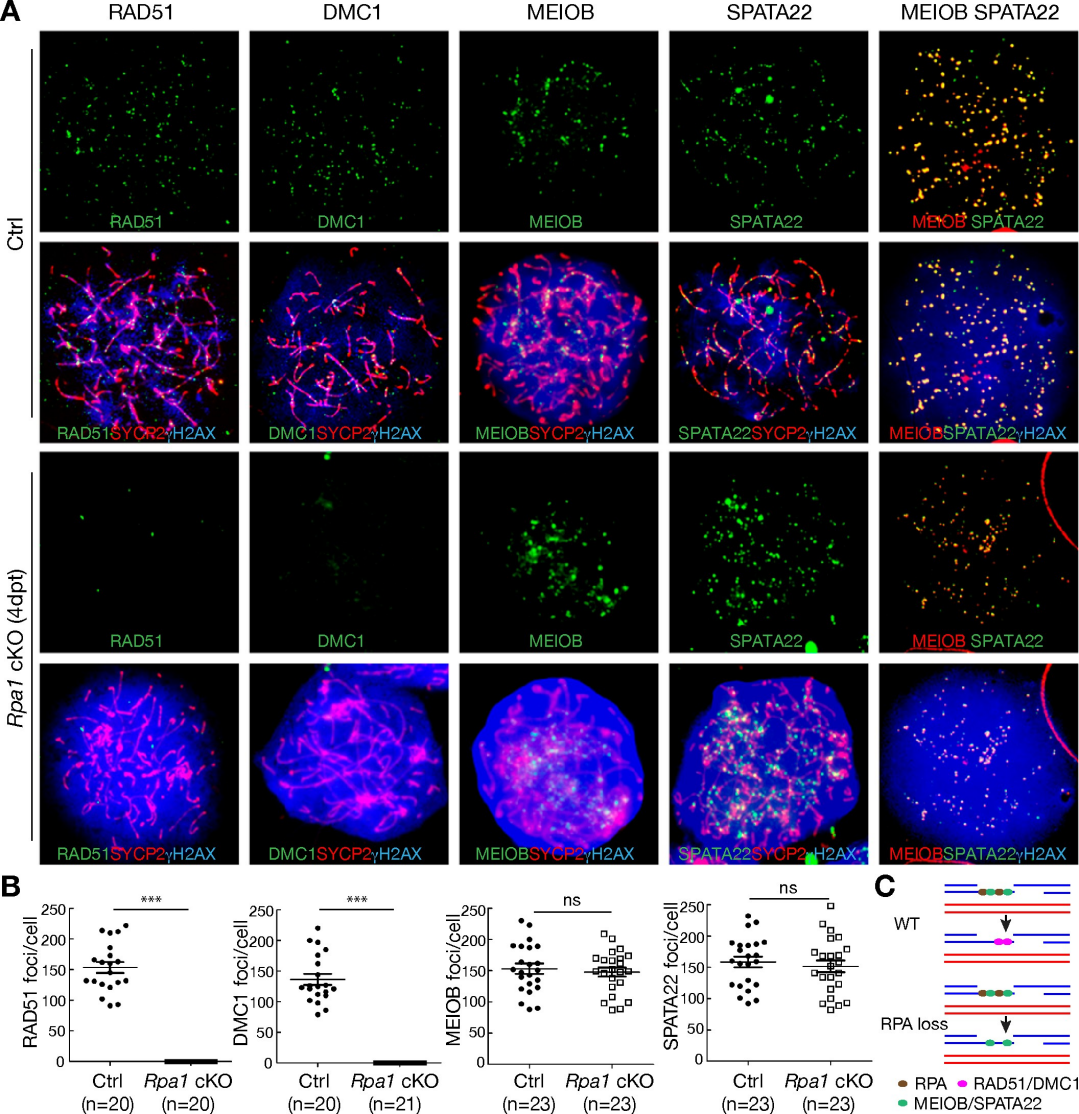
RPA1 is required for focal localization of RAD51/DMC1 on meiotic chromosomes but not MEIOB/SPATA22 at the zygotene stage. (A) Immunolocalization of ssDNA-binding proteins in zygotene (control) and zygotene-like (*Rpa1*^fl/fl^ *Ddx4-*Cre^ERT2^, 4 dpt) spermatocytes. Synaptonemal complexes were immunostained with anti-SYCP2 antibody. Note that γH2AX signal is much stronger in mutant zygotene-like spermatocytes than control zygonema. (B) Dot plots of foci of RAD51, DMC1, MEIOB, and SPATA22 in zygotene (control) and zygotene-like (*Rpa1*^cKO^) spermatocytes. Solid lines show the average ± SD. n, number of cells counted from two experiments; ***, p < 0.001 (Student’s *t* test); ns, non-significant. (C) A role for RPA in pre-synaptic filament formation. Upper panel, control; bottom panel, absence of RPA.

During meiotic recombination, DNA recombinases RAD51 and DMC1 form nuclear complexes on meiotic chromatin [10, 11] and generate presynaptic filaments on ssDNA that drive strand invasion into homologous DNA duplex [23, 24]. While RAD51 and DMC1 formed foci on meiotic chromosomes in wild type mice (Fig 5A), such foci were surprisingly absent in *Rpa1*-deficient zygotene-like spermatocytes (Fig 5A and 5B), although both proteins were present at reduced abundance in the mutant testes as shown by Western blotting (Fig 2D). This result raises the possibility that RPA binding to ssDNA precedes and is a pre-requisite for loading of RAD51 and DMC1 recombinases (Fig 5C). In combination with our colocalization data (Fig 1), our results suggest that the interaction between RPA and RAD51/DMC1 is transient and that RPA is replaced by RAD51/DMC1 in the foci that form on DSBs. As such, loss of RPA blocks the critical strand invasion step in meiotic recombination and leads to a complete failure in chromosomal synapsis as observed in zygotene-like spermatocytes (Fig 5C).

ATR promotes loading of RAD51 and DMC1 to DSBs during meiotic recombination [25, 26]. In wild type spermatocytes, ATR formed foci on the synaptonemal complex at the zygotene stage and localized to the XY body at the pachytene stage (S4 Fig), consistent with the previous reports [26–28]. In pachytene spermatocytes from *Rpa1*^cKO^ testes (4 dpt), ATR still localized to the XY body (S4B Fig). In addition, ATR still formed foci in zygotene-like *Rpa1* mutant spermatocytes (S4A Fig). These results show that ATR is not sufficient for loading of RAD51 and DMC1 to DSBs in the absence of RPA.

### Localization of MEIOB/SPATA22 to DSBs is independent of RPA

We next assessed if MEIOB contributes to RPA-dependent regulation of meiotic recombination. In wild type mice, the MEIOB/SPATA22 dimer always colocalized with RPA in foci on meiotic chromosomes (Fig 1H) [13]. In sharp contrast with RAD51/DMC1, MEIOB and SPATA22 still co-localized in *Rpa1*-deficient zygotene-like spermatocytes, suggesting that the formation and loading of MEIOB/SPATA22 dimers on DSBs are independent of RPA (Fig 5). In addition, this result demonstrates that MEIOB/SPATA22 dimers are insufficient to recruit RAD51/DMC1 to DSBs (Fig 5C). Taken together with the previous finding on the presence of RPA foci in *Meiob*-deficient spermatocytes [13], the RPA trimer and the MEIOB/SPATA22 dimer localize to DSBs independently and function non-redundantly in meiotic recombination.

### RPA promotes crossover formation

Following strand invasion, a subset of DSBs are repaired into crossovers at the pachytene stage. At the subsequent diplotene and metaphase stages, crossover sites become chiasmata to physically link bivalent homologs till chromosome segregation. Strand invasion into the homologue duplex creates the displacement loop (D-loop), which is single stranded. RPA formed foci in early-to-mid pachynema, presumably binding to the D loop and the single stranded second end (Fig 1). We first examined the RPA depletion efficiency in pachytene spermatocytes at 2 dpt and 6 dpt by nuclear spread analysis. At 2 dpt, the number of RPA2 foci in early-mid pachytene spermatocytes was comparable between control and *Rpa1*^cKO^ testes (S5 Fig), even though the RPA2 protein abundance was reduced in *Rpa1*^cKO^ testes (Fig 2D). However, at 6 dpt, in contrast with control early-mid pachytene spermatocytes, foci of RPA1, RPA2, or RPA3 were dramatically reduced in number and intensity in the early-mid pachytene mutant spermatocytes (S6 Fig). Therefore, to investigate whether RPA plays a role in crossover formation, we analysed pachytene spermatocytes from *Rpa1*^cKO^ testes at 6 or 8 days post-tamoxifen treatment.

We examined proteins involved in meiotic recombination that form foci at the pachytene stage. MEIOB and SPATA22 foci were still present, but RAD51 and DMC1 foci were absent in *Rpa1*-deficient early-mid pachytene spermatocytes (S7 Fig). This localization result was similar to that in zygotene-like *Rpa1*-deficient spermatocytes (Fig 5), confirming the unique role of RPA in RAD51/DMC1 localization throughout meiosis.

**Fig 6:**
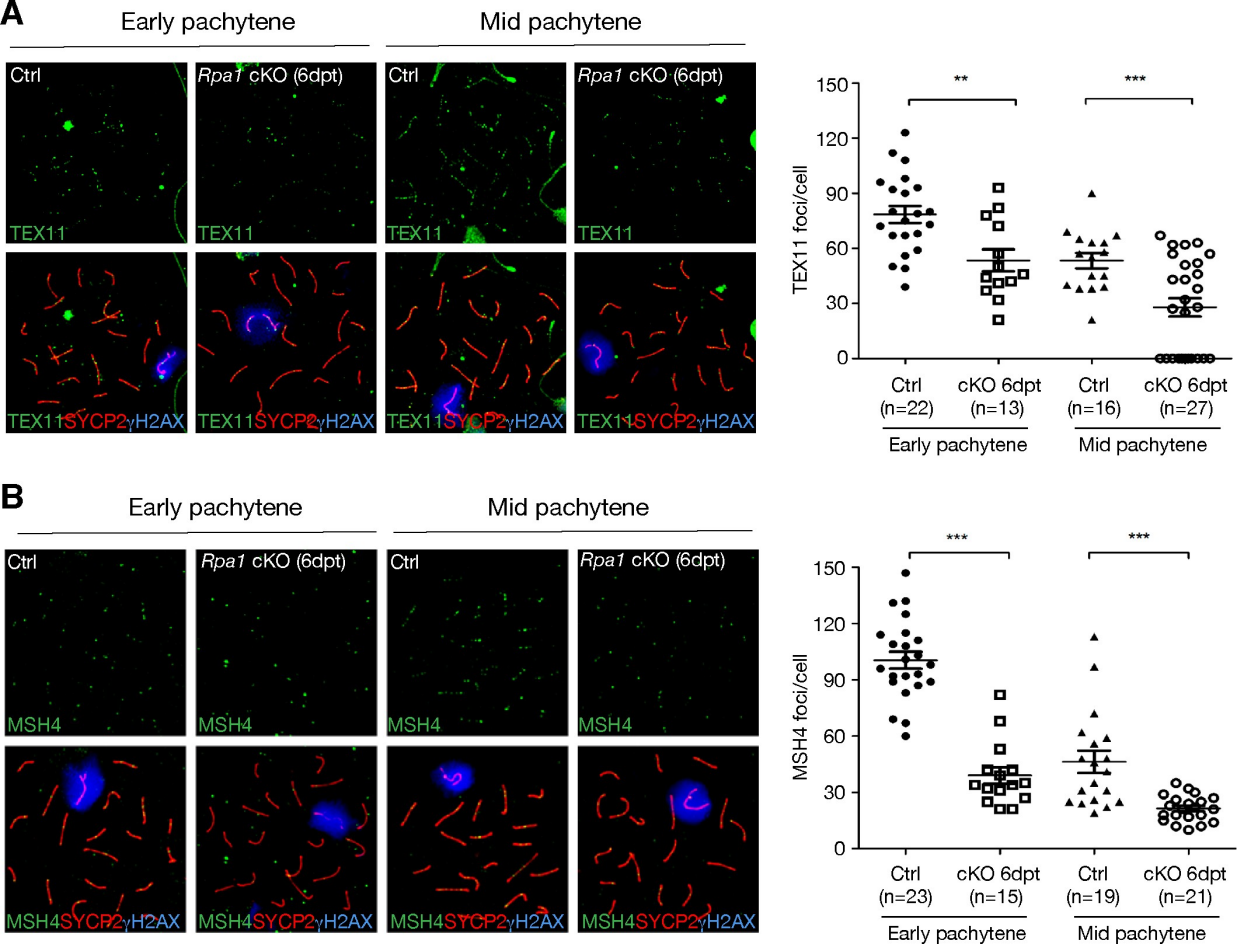
Reduction in the number of TEX11 and MSH4 foci in early/mid pachytene spermatocytes from *Rpa1*^cKO^ mice at 6 days post-tamoxifen treatment. Spermatocytes from untreated adult males were used as controls. The number of TEX11 foci (A) or MSH4 foci (B) in mid-pachytene spermatocytes is lower than in early pachytene spermatocytes. Early and mid-pachytene spermatocytes were categorized by the morphology of XY chromosomal axis and the intensity of the synaptonemal complex staining as previously described [41]. Early pachytene stage is characterized by an extended XY configuration and low intensity of the synaptonemal complex staining, whereas the mid-pachytene spermatocytes exhibit a U-shaped XY axis and higher synaptonemal complex staining. n, number of spermatocytes counted from four experiments (A) or three experiments (B); **, p< 0.01; ***, p<0.001.

We next analysed two meiosis-specific factors, TEX11 and MSH4 [29–31], which modulate crossover formation. These two factors formed foci in control early-to-mid pachytene spermatocytes (Fig 6). However, the number of TEX11 foci was significantly reduced in early-mid pachytene spermatocytes from *Rpa1* cKO testes at 6 dpt (Fig 6A). Likewise, the number of MSH4 foci decreased significantly (Fig 6B). The reduction in the number of TEX11 and MSH4 foci in *Rpa1*-deficient pachynema (Fig 6) suggests a role of RPA in crossover formation.

**Fig 7:**
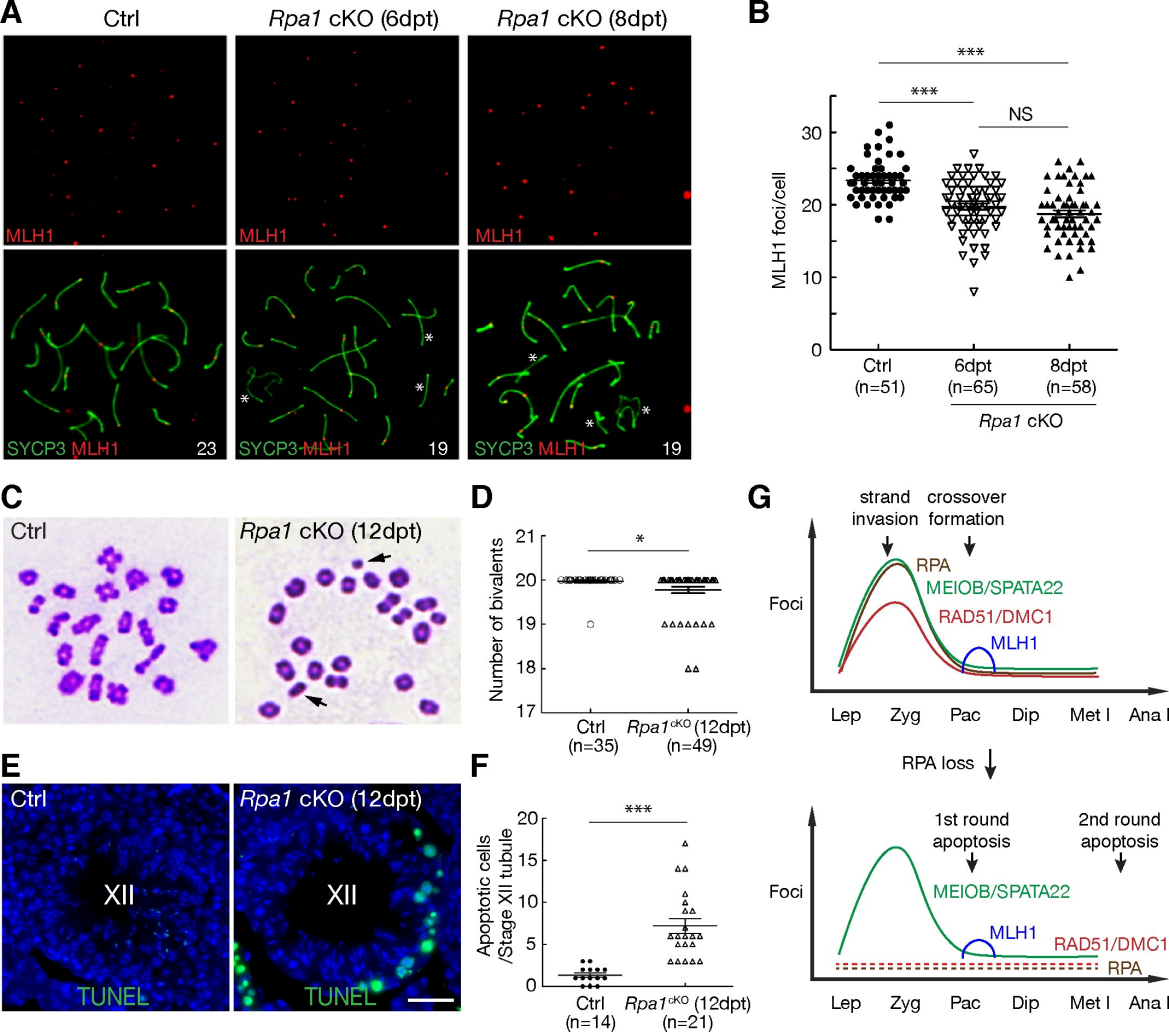
RPA1 regulates the formation of meiotic crossovers. (A) Reduction in MLH1 foci in *Rpa1*^cKO^ pachytene spermatocytes. Asterisks mark the chromosomes without MLH1 foci. (B) Counts of MLH1 foci in pachytene spermatocytes. n, number of cells counted from four experiments. (C) Presence of univalent chromosomes (indicated by arrows) in *Rpa1*^cKO^ metaphase I spermatocytes. (D) Quantification of chiasmata from control and *Rpa1*^cKO^ metaphase I cells. n, number of cells counted from three experiments. (E) TUNEL analysis of stage XII seminiferous tubules from adult control and *Rpa1*^cKO^ males. Note the TUNEL-positive (green) metaphase I spermatocytes in the *Rpa1*^cKO^ tubule. Scale bar, 25 μm. (F) Quantification of apoptotic cells of stage XII tubules from adult control and *Rpa1*^cKO^ males. n, number of stage XII tubules from two experiments. ns, no significance; ***, p ≤ 0.001 (Student’s *t*-Test); dpt, days post tamoxifen injection. (G) Dual functions of RPA in meiotic recombination. The upper diagram illustrates the distribution of recombination proteins and timing of key recombination events during male meiosis. The lower diagram depicts the defects in the absence of RPA: depletion of RAD51/DMC1, reduction in MLH1 foci, and two rounds of apoptosis (the first round at stage IV and the second round at stage XII). Abbreviations: Lep, leptotene spermatocytes; Zyg, zygotene; Pac, pachytene; Dip, diplotene; Met I, metaphase I; Ana I, anaphase I.

To directly assess the effect of RPA on crossovers, we immunostained for MLH1, which localizes specifically to future crossover sites. MLH1 foci were present but significantly reduced in number in *Rpa1*-deficient pachynema compared with control at both 6 dpt and 8 dpt (Fig 7A and 7B). Analysis of metaphase nuclei revealed that 20% of metaphase I spermatocytes had univalent chromosomes in *Rpa1*^cKO^ testes (Fig 7C and 7D). Univalents were observed for both autosomes and sex chromosomes. The presence of univalent chromosomes leads to chromosome mis-segregation and triggers the spindle checkpoint (Fig 3D). Indeed, we detected massive apoptosis of spermatocytes in stage XII seminiferous tubules from *Rpa1*^cKO^ testes, which contained metaphase and anaphase spermatocytes (Figs 7E, 7F and 3A—12 dpt). These results demonstrate that RPA promotes crossover formation and thus is required for proper chromosome segregation during male meiosis (Fig 7G).

## Discussion

RPA is often included in *in vitro* recombination reactions but its specific requirement is unknown. Recombinases RAD51 and DMC1 form helical nucleoprotein filaments on ssDNA [7, 32] and RPA is necessary for efficient RAD51 filament formation [32]. The DNA strand exchange activities catalysed by RAD51 or DMC1 nucleoprotein filaments are rather inefficient but strongly stimulated by RPA [6, 7, 32]. These biochemical studies support RPA as an important accessary factor in recombination. While studies of yeast hypomorphic *Rpa1* mutants also support a role for RPA in meiotic recombination [33], these studies did not address whether RPA is required for pre-synaptic filament formation. Using loss of function *Rpa1* mouse mutants, we demonstrate that RPA loading to meiotic DSBs is a pre-requisite for RAD51/DMC1 loading *in vivo* (Fig 5C). One possible explanation is that RPA loading prevents formation of ssDNA secondary structure and recruits RAD51/DMC1 to these sites of recombination. An alternative but less likely explanation is that RAD51/DMC1 nucleation on ssDNA is independent of RPA, but that their localization to DSBs is stabilized by RPA. Finally, the fact that MEIOB/SPATA22 still localizes as foci in *Rpa1*-null spermatocytes suggests that the requirement of RPA in RAD51/DMC1 loading is unique and cannot be compensated for by other ssDNA-binding complexes such as MEIOB/SPATA22.

MEIOB, SPATA22, and RPA colocalize in foci on meiotic chromosomes [13, 34]. In *Meiob* or *Spata22*-deficient germ cells, RPA foci are still present [13, 14, 34, 35]. Here we find that MEIOB and SPATA22 still form foci in RPA1-deficient spermatocytes. Collectively, although the MEIOB/SPATA22 dimer and the RPA trimer colocalize in foci and interact with each other [15], their localizations to DSBs are independent. A previous study suggests that SPATA22 is required for the maintenance but not formation of RAD51 foci in rat spermatocytes [35]. RAD51 and DMC1 foci are present in *Meiob*-deficient spermatocytes [13]. Therefore, the presence of MEIOB/SPATA22 foci and the absence of RAD51/DMC1 foci in RPA-deficient spermatocytes support that RPA but not MEIOB/SPATA22 is required for formation of RAD51/DMC1 foci.

Our genetic study identifies an essential role for RPA in meiotic recombination—strand invasion at the zygotene stage (Fig 7G). Failure in strand invasion causes persistence of DSBs and DNA damage in early *Rpa1*-deficient zygotene-like spermatocytes, which are eliminated by the first round of apoptosis in stage IV seminiferous tubules due to the pachytene checkpoint (Fig 3D, Fig 7G and S3 Fig). Therefore, *Rpa1*-deficient zygotene-like spermatocytes would not be expected to progress through the pachytene stage.

Our study uncovers a second role for RPA in meiotic recombination—crossover formation at the pachytene stage (Fig 7G). We postulated that the pachytene spermatocytes from *Rpa1*^cKO^ testes were already at the pachytene stage and lost RPA1 upon tamoxifen injection, and thus were not likely to be derived from the defective zygotene-like spermatocytes. Inactivation of RPA1 in pachytene spermatocytes reduces crossover formation but does not completely abrogate it. This partial effect was likely due to the conditional deletion approach. RPA was dramatically depleted in *Rpa1*^cKO^ pachytene spermatocytes at 6 dpt, but was still detectable as extremely weak foci at a greatly reduced number (S6 Fig). A second possibility is that the existing RPA1 protein may have played a role in crossover formation before being degraded. Lastly, MEIOB/SPATA22 may exert a more important role in crossover formation than does RPA, since MEIOB/SPATA22 still forms foci in *Rpa1*-deficient pachytene spermatocytes. We hypothesize that, in the absence of RPA, MEIOB/SPATA22 binds to the recombination intermediates to promote the formation of most but not all crossovers (Fig 7G). Regardless of these scenarios, reduction in crossover formation in *Rpa1*-deficient pachytene spermatocytes leads to chromosome mis-segregation at the anaphase I stage spermatocytes, which are depleted by the second round of apoptosis due to the spindle checkpoint activation (Figs 3D and 7G).

Previously, a knockdown approach was employed to investigate the function of an essential gene *Rad51* in mouse spermatogenesis [36]. In this study, we have used a powerful inducible germline-specific inactivation strategy to elucidate the function of RPA, an essential gene involved in DNA metabolism. This approach can be used to study the functions of any somatically essential genes in meiosis. Our results demonstrate that RPA plays a dual function in meiotic recombination: an essential role in presynaptic filament formation and an important role in crossover formation.

## Materials and methods

### Ethics statement

Mice were maintained and used for experimentation according to the protocol approved by the Institutional Care and Use Committee of the University of Pennsylvania.

### Mice

*Rpa1*^tm1a^ mice (EUCOMM consortium) [37] were mated with ROSA26-FLPo mice (Stock number: 007844, Jackson Laboratory) [38] to generate a floxed (*Rpa1*^fl^) allele (Fig 2). *Rpa1*^fl/fl^ females were crossed with *Ddx4-*Cre^ERT2^ males (Stock number: 024760, Jackson Laboratory) to generate *Rpa1*^fl/+^ *Ddx4-*Cre^ERT2^ mice. *Rpa1*^fl/+^ *Ddx4-*Cre^ERT2^ males were crossed with *Rpa1*^fl/fl^ females to obtain *Rpa1*^fl/fl^ *Ddx4-*Cre^ERT2^ mice. *Rpa1*^fl/fl^ *Ddx4-*Cre^ERT2^ mice were fertile and thus were used to breed with *Rpa1*^fl/fl^ mice to produce more *Rpa1*^fl/fl^ *Ddx4-*Cre^ERT2/+^ mice for experiments. To generate germ cell-specific *Rpa1* knockout mice, injection of tamoxifen was used to induce Cre-mediated deletion of the floxed exon. In brief, tamoxifen (Sigma, T5648) was dissolved in corn oil (Sigma, C8267) at a concentration of 20 mg/ml and injected intraperitoneally into the 8-week-old *Rpa1*^fl/fl^ *Ddx4-*Cre^ERT2/+^ *male* mice at a dose of 4 mg/30g body weight for five consecutive days. Untreated *Rpa1*^fl/fl^ *Ddx4-*Cre^ERT2/+^ littermates were used as controls. Tamoxifen treatment in combination with *Ddx4-*Cre^ERT2^ in otherwise wild type males does not adversely affect meiosis as previously reported [39]. *Ddx4*-Cre (Stock number: 006954) and *Stra8*-Cre (Stock number: 008208) mice were obtained from the Jackson laboratory. Mice were genotyped by PCR analysis of tail genomic DNA. Wild-type allele (264 bp) and *Rpa1* floxed allele (277 bp) was assayed by PCR with primers GATTATGACACCCTTTGGGACT and TGGCCAAATTAAACCACAGTAACACG. *Ddx4-*Cre^ERT2^ allele (~205 bp) was assayed by PCR with primers ATACCGGAGATCATGCAAGC and GGCCAGGCTGTTCTTCTTAG.

### Antibody production

The mouse RPA2 full-length and RPA3 full-length were expressed as 6xHis-RPA2 and 6xHis-RPA3 fusion proteins in *E*. *coli* using the pQE-30 vector and affinity purified with Ni-NTA agarose. Two rabbits were immunized with each fusion protein (Cocalico Biologicals Inc.). Two guinea pigs were also immunized with the RPA2 recombinant protein. The resulting antisera UP2436 (rabbit anti-RPA2), GP111 (guinea pig anti-RPA2), and UP2439 (anti-RPA3) were used for immunofluorescence and western blotting analyses.

### Histological analysis and TUNEL assays

For histological analysis, testes were fixed in Bouin’s solution, embedded with paraffin, and sectioned. Sections were stained with hematoxylin and eosin. For TUNEL analysis, testes were fixed in 4% paraformaldehyde overnight at 4°C, dehydrated in 30% sucrose and sectioned. Sections were performed with the TUNEL Enzyme and Label Kit (Roche Boehringer Mannheim).

### Surface nuclear spread analyses

Nuclear spread analysis of spermatocytes was performed as previously described [40]. Primary antibodies that were used for immunofluorescence were listed in S1 Table. For quantification of foci, images of spermatocytes from two to four animals were captured and analysed. Axial element markers SYCP2 or SYCP3 were used to classify the stage of meiotic prophase on nuclear spreads [43, 44]. Early and mid-pachytene spermatocytes were distinguished by the morphology of XY chromosomal axis and the intensity of synaptonemal complex [41]. Characteristics of early pachynema: relatively low intensity of synaptonemal complex staining, short synapsed pseudoautosomal regions, and/or unsynapsed ends of a few autosomes. Characteristics of mid pachynema: strong intensity of synaptonemal complex staining, full synapsis of all autosomal pairs, and often U-shaped XY axis. Characteristics of late pachynema: accumulation of SYCP2/3 proteins at the synaptonemal complex ends and figure -8 shaped XY chromosomes. The abnormal zygotene-like spermatocytes from *Rpa1* mutant testes were characterized by extremely strong γH2AX signals and clusters of ends of axial elements. Metaphase spread cells were stained with 4% Gurr Giemsa.

### Immunoblotting assays

For Western blot analysis, testes were homogenized in 500 μl protein extraction buffer (62.5 mM Tris-HCl (pH 6.8), 3% SDS, 10% glycerol, 5% 2-mercaptoethanol). Samples were boiled in 2x loading buffer for 10–15 min to obtain soluble testicular protein extracts. About 10–20 μl of testicular extracts were resolved by SDS-PAGE, transferred onto nitrocellulose membranes using iBlot (Invitrogen), and immunoblotted with indicated antibodies (S1 Table).

### Imaging

Color histological images were captured on a Leica DM5500B microscope with a DFC450 digital color camera (Leica Microsystems). Immunolabeled chromosome spreads and testis TUNEL assay images were captured with an ORCA Flash4.0 digital monochrome camera (Hamamatsu Photonics) on a Leica DM5500B microscope (Leica Microsystems) and processed using Photoshop (Adobe) software packages. Super-resolution imaging microscopy analysis was performed using a Nikon NSIM super-resolution microscope system and NIS-Elements 2 image processing software.

### BrdU incorporation assay

Adult control (wild type) and *Rpa1*^cKO^ male mice were intraperitoneally injected with 50 mg/kg body weight of BrdU (Sigma, B5002). Adult *Rpa1*^fl/fl^ *Ddx4-*Cre^ERT2/+^ male mice were treated with daily injection of tamoxifen for five consecutive days as described above. 12 hours after the last tamoxifen injection, a single dose of BrdU was injected. After 2 h, the mice were sacrificed. Testes were collected and fixed in the fixative solution (30% formaldehyde, 15% ethyl alcohol, 5% glacial acetic acid) overnight, embedded in paraffin, and cut in 5 μm sections. After deparaffinization and rehydration, slides were immersed in 20 mM Tris-HCl, pH9.0, at 95°C for 15 min. The slides were blocked with 1% BSA for 1h at room temperature followed by incubating with anti-BrdU and anti-SP10 antibodies at 37°C overnight. The morphology of acrosome revealed by anti-SP10 (ACRV1) antibody was used to precisely identify stage VII-VIII tubules, in which preleptotene spermatocytes are present [42]. Slides were rinsed with PBS and then incubated with rabbit anti-rat (Vector, FI-4001) and goat anti-guinea pig secondary antibodies (Novus, NB-1206906) at 37°C for 1h. Mounting medium with DAPI was added to the slides for imaging. Three pairs of control and *Rpa1*^cKO^ males (0 day post tamoxifen treatment) were analyzed. At least thirty five stage VII-VIII tubules were counted for each mouse (Fig 4B and 4C).

### RT-PCR expression analysis of *Rpa1*

Adult wild type and *Rpa1*^fl/fl^ *Ddx4-*Cre^ERT2/+^ male mice were treated with daily injection of tamoxifen for five consecutive days as described above. Testes were collected at 2 dpt, 4 dpt, and 6 dpt for semi-quantitative RT-PCR analysis (S2 Fig). The pre-exon 8 *Rpa1* region was assayed by RT-PCR (212 bp) using primers GAACACGCTTTCCTCGTTCATGCTG (exons 3/4) and CTTCATTATAGGGCACTGGATTCCC (exon 6). The post-exon 8 *Rpa1* region was assayed by RT-PCR (256 bp) using primers AGAGCTACTGCTTTCAATGAGCAAG (exon 10) and TGTCTACTAGTGCGTCTTTAGCCTT (exons 11/12). *Actb* (382 bp) was assayed with primers AGAAGAGCTATGAGCTGCCT and TCATCGTACTCCTGCTTGCT.

### Statistics

Statistical analysis was performed with Student's *t*-test.

## Supporting information

S1 Fig Histological analysis of testes from adult *Rpa1*^fl/-^ *Ddx4*-Cre and *Rpa1*^fl/-^ *Stra8*-Cre males. (A) Complete loss of germ cells in testes from 10-week-old *Rpa1*^fl/-^ *Ddx4*-Cre males. All seminiferous tubules in the *Rpa1*^cKO^ males are Sertoli cell only. *Rpa1*^fl/-^ males are controls. (B) Heterogeneity of testicular histology in 8-week-old *Rpa1*^f/-^ *Stra8*-Cre males. Like the wild type control, some seminiferous tubules in the *Rpa1* mutant testis have a full spectrum of germ cells, including spermatocytes and spermatids, possibly due to a lack of or inefficient *Stra8*-Cre-mediated *Rpa1* deletion. Other seminiferous tubules in the *Rpa1* mutant testis are nearly devoid of all germ cells (marked by asterisks), possibly due to the expression of *Stra8*-Cre in spermatogonia. Scale bars, 50 μm.
(TIF)

S2 Fig Expression analysis of *Rpa1* transcript in adult wild type and *Rpa1*^fl/fl^ *Ddx4-*Cre^ERT2/+^ testes. Exon 8 in *Rpa1*^fl^ allele is flanked by loxP sites (Fig 2A). Deletion of exon 8 results in a frame shift in the resulting *Rpa1* mutant transcript. Two RT-PCR assays were designed to amplify the pre-exon 8 region (A) and the post-exon 8 region (B) of *Rpa1* transcript. *Actb* serves a control. dpt, days post-tamoxifen treatment.
(TIF)

S3 Fig Increased apoptosis in testes from *Rpa1*^cKO^ mice at 4 days post-tamoxifen treatment. TUNEL analysis was performed on frozen testicular sections from adult control and *Rpa1*^cKO^ mice at 4 days post-tamoxifen treatment. The seminiferous tubule stage is shown in the middle of the panels. Apoptotic cells are shown in green. DNA was stained with DAPI. Dramatically increased apoptosis in spermatocytes occurs specifically in stage IV *Rpa1*^cKO^ seminiferous tubules, which correspond to the pachytene checkpoint during male meiosis. Scale bar, 25 μm.
(TIF)

S4 Fig ATR localization is independent of RPA1 in spermatocytes. Immunolocalization of ATR was performed in spermatocytes from control and *Rpa1*^cKO^ testes (4 days post-tamoxifen treatment) at the zygotene and zygotene-like stages (A) and the pachytene stage (B).
(TIF)

S5 Fig Analysis of RPA2 foci in control and *Rpa1*^cKO^ spermatocytes at 2 days post-tamoxifen treatment (dpt). Among three RPA subunits, immunofluorescence of RPA2 on surface spread of spermatocyte nuclei was the strongest. Therefore, RPA2 immunostaining was performed and RPA foci were counted. (A) Presence of RPA2 foci in leptotene, zygotene, and early-mid pachytene spermatocytes from both control and *Rpa1*^cKO^ mice at 2 dpt. Scale bar, 25 μm. (B) Quantification of RPA2 foci in control and *Rpa1*^cKO^ spermatocytes. n, number of spermatocytes; ns, not statistically significant.
(TIF)

S6 Fig Reduction of RPA1, RPA2, and RPA3 foci in *Rpa1*^cKO^ pachytene spermatocytes at 6 days post-tamoxifen treatment (dpt). (A) RPA1, RPA2, and RPA3 form foci on the synaptonemal complex in control early-mid pachytene spermatocytes (top panels) but the RPA foci are sharply reduced in number and intensity in *Rpa1*^cKO^ early-mid pachytene spermatocytes (bottom panels). Scale bar, 25 μm. (B) Quantification of RPA2 foci in control and *Rpa1*^cKO^ (6 dpt) early-mid pachytene spermatocytes. n, number of early-mid pachytene spermatocytes.
(TIF)

S7 Fig RPA1 is required for focal localization of RAD51/DMC1 on meiotic chromosomes but not MEIOB/SPATA22 at the pachytene stage. (A) Immunolocalization of ssDNA-binding proteins in control and *Rpa1*^cKO^ early/mid- pachytene (6 days post-tamoxifen treatment) spermatocytes. Synaptonemal complexes were immunostained with anti-SYCP2 antibody. Only XY body is γH2AX-positive. (B) Dot plots of foci of RAD51, DMC1, MEIOB, and SPATA22 in control and *Rpa1*^cKO^ early/mid pachytene spermatocytes. Solid lines show the average ± SD. n, number of cells counted from two experiments; ***, p < 0.001 (Student’s *t* test); ns, non-significant.
(TIF)

S1 Table Primary antibodies used in the study. (DOCX)

S2 Table Numerical data that underlies graphs. (XLSX)

## References

[1] Handel MA, Schimenti JC. (2010) Genetics of mammalian meiosis: Regulation, dynamics and impact on fertility. Nat Rev Genet 11: 124–136. 10.1038/nrg2723 20051984

[2] Hunter N. (2015) Meiotic recombination: The essence of heredity. Cold Spring Harb Perspect Biol 7: 10.1101/cshperspect.a016618 26511629 PMC4665078

[3] Hassold T, Hunt P. (2001) To err (meiotically) is human: The genesis of human aneuploidy. Nat Rev Genet 2: 280–291. 10.1038/35066065 11283700

[4] Shinohara A, Ogawa H, Ogawa T. (1992) Rad51 protein involved in repair and recombination in S. cerevisiae is a RecA-like protein. Cell 69: 457–470. 1581961 10.1016/0092-8674(92)90447-k

[5] Bishop DK, Park D, Xu L, Kleckner N. (1992) DMC1: A meiosis-specific yeast homolog of E. coli recA required for recombination, synaptonemal complex formation, and cell cycle progression. Cell 69: 439–456. 1581960 10.1016/0092-8674(92)90446-j

[6] Sung P. (1994) Catalysis of ATP-dependent homologous DNA pairing and strand exchange by yeast RAD51 protein. Science 265: 1241–1243. 8066464 10.1126/science.8066464

[7] Sehorn MG, Sigurdsson S, Bussen W, Unger VM, Sung P. (2004) Human meiotic recombinase Dmc1 promotes ATP-dependent homologous DNA strand exchange. Nature 429: 433–437. 10.1038/nature02563 15164066

[8] Wold MS. (1997) Replication protein A: A heterotrimeric, single-stranded DNA-binding protein required for eukaryotic DNA metabolism. Annu Rev Biochem 66: 61–92. 10.1146/annurev.biochem.66.1.61 9242902

[9] Golub EI, Gupta RC, Haaf T, Wold MS, Radding CM. (1998) Interaction of human rad51 recombination protein with single-stranded DNA binding protein, RPA. Nucleic Acids Res 26: 5388–5393. 9826763 10.1093/nar/26.23.5388 PMC148005

[10] Bishop DK. (1994) RecA homologs Dmc1 and Rad51 interact to form multiple nuclear complexes prior to meiotic chromosome synapsis. Cell 79: 1081–1092. 7528104 10.1016/0092-8674(94)90038-8

[11] Tarsounas M, Morita T, Pearlman RE, Moens PB. (1999) RAD51 and DMC1 form mixed complexes associated with mouse meiotic chromosome cores and synaptonemal complexes. J Cell Biol 147: 207–220. 10525529 10.1083/jcb.147.2.207 PMC2174216

[12] Petukhova GV, Pezza RJ, Vanevski F, Ploquin M, Masson JY, et al (2005) The Hop2 and Mnd1 proteins act in concert with Rad51 and Dmc1 in meiotic recombination. Nat Struct Mol Biol 12: 449–453. 10.1038/nsmb923 15834424

[13] Luo M, Yang F, Leu NA, Landaiche J, Handel MA, et al (2013) MEIOB exhibits single-stranded DNA-binding and exonuclease activities and is essential for meiotic recombination. Nat Commun 4: 2788 10.1038/ncomms3788 24240703 PMC3891831

[14] Souquet B, Abby E, Herve R, Finsterbusch F, Tourpin S, et al (2013) MEIOB targets single-strand DNA and is necessary for meiotic recombination. PLoS Genet 9: e1003784 10.1371/journal.pgen.1003784 24068956 PMC3778009

[15] Xu Y, Greenberg RA, Schonbrunn E, Wang PJ. (2017) Meiosis-specific proteins MEIOB and SPATA22 cooperatively associate with the ssDNA-binding RPA complex and DNA double-strand breaks. Biol Reprod 96: 1096–1104. 10.1093/biolre/iox040 28453612 PMC6355104

[16] Ribeiro J, Abby E, Livera G, Martini E. (2016) RPA homologs and ssDNA processing during meiotic recombination. Chromosoma 125: 265–276. 10.1007/s00412-015-0552-7 26520106 PMC4830875

[17] Wang Y, Putnam CD, Kane MF, Zhang W, Edelmann L, et al (2005) Mutation in Rpa1 results in defective DNA double-strand break repair, chromosomal instability and cancer in mice. Nat Genet 37: 750–755. 10.1038/ng1587 15965476

[18] Moens PB, Kolas NK, Tarsounas M, Marcon E, Cohen PE, et al (2002) The time course and chromosomal localization of recombination-related proteins at meiosis in the mouse are compatible with models that can resolve the early DNA-DNA interactions without reciprocal recombination. J Cell Sci 115: 1611–1622. 11950880 10.1242/jcs.115.8.1611

[19] Gallardo T, Shirley L, John GB, Castrillon DH. (2007) Generation of a germ cell-specific mouse transgenic cre line, vasa-cre. Genesis 45: 413–417. 10.1002/dvg.20310 17551945 PMC2597027

[20] Sadate-Ngatchou PI, Payne CJ, Dearth AT, Braun RE. (2008) Cre recombinase activity specific to postnatal, premeiotic male germ cells in transgenic mice. Genesis 46: 738–742. 10.1002/dvg.20437 18850594 PMC2837914

[21] Bao J, Ma HY, Schuster A, Lin YM, Yan W. (2013) Incomplete cre-mediated excision leads to phenotypic differences between Stra8-iCre; Mov10l1(lox/lox) and Stra8-iCre; Mov10l1(lox/Delta) mice. Genesis 51: 481–490. 10.1002/dvg.22389 23554062 PMC3918465

[22] John GB, Gallardo TD, Shirley LJ, Castrillon DH. (2008) Foxo3 is a PI3K-dependent molecular switch controlling the initiation of oocyte growth. Dev Biol 321: 197–204. 10.1016/j.ydbio.2008.06.017 18601916 PMC2548299

[23] Masson JY, West SC. (2001) The Rad51 and Dmc1 recombinases: A non-identical twin relationship. Trends Biochem Sci 26: 131–136. 11166572 10.1016/s0968-0004(00)01742-4

[24] Brown MS, Bishop DK. (2014) DNA strand exchange and RecA homologs in meiosis. Cold Spring Harb Perspect Biol 7: a016659 10.1101/cshperspect.a016659 25475089 PMC4292170

[25] Pacheco S, Maldonado-Linares A, Marcet-Ortega M, Rojas C, Martinez-Marchal A, et al (2018) ATR is required to complete meiotic recombination in mice. Nat Commun 9: 2622-018-04851-z. 10.1038/s41467-018-04851-z PMC6033890 29977027

[26] Widger A, Mahadevaiah SK, Lange J, ElInati E, Zohren J, et al (2018) ATR is a multifunctional regulator of male mouse meiosis. Nat Commun 9: 2621-018-04850-0. 10.1038/s41467-018-04850-0 PMC6033951 29976923

[27] Royo H, Prosser H, Ruzankina Y, Mahadevaiah SK, Cloutier JM, et al (2013) ATR acts stage specifically to regulate multiple aspects of mammalian meiotic silencing. Genes Dev 27: 1484–1494. 10.1101/gad.219477.113 23824539 PMC3713429

[28] Ichijima Y, Ichijima M, Lou Z, Nussenzweig A, Camerini-Otero RD, et al (2011) MDC1 directs chromosome-wide silencing of the sex chromosomes in male germ cells. Genes Dev 25: 959–971. 10.1101/gad.2030811 21536735 PMC3084029

[29] Yang F, Gell K, van der Heijden GW, Eckardt S, Leu NA, et al (2008) Meiotic failure in male mice lacking an X-linked factor. Genes Dev 22: 682–691. 10.1101/gad.1613608 18316482 PMC2259036

[30] Yang F, Silber S, Leu NA, Oates RD, Marszalek JD, et al (2015) TEX11 is mutated in infertile men with azoospermia and regulates genome-wide recombination rates in mouse. EMBO Mol Med 7: 1198–1210. 10.15252/emmm.201404967 26136358 PMC4568952

[31] Kneitz B, Cohen PE, Avdievich E, Zhu L, Kane MF, et al (2000) MutS homolog 4 localization to meiotic chromosomes is required for chromosome pairing during meiosis in male and female mice. Genes Dev 14: 1085–1097. 10809667 PMC316572

[32] Sugiyama T, Zaitseva EM, Kowalczykowski SC. (1997) A single-stranded DNA-binding protein is needed for efficient presynaptic complex formation by the saccharomyces cerevisiae Rad51 protein. J Biol Chem 272: 7940–7945. 9065463 10.1074/jbc.272.12.7940

[33] Soustelle C, Vedel M, Kolodner R, Nicolas A. (2002) Replication protein A is required for meiotic recombination in saccharomyces cerevisiae. Genetics 161: 535–547. 12072452 10.1093/genetics/161.2.535 PMC1462150

[34] Hays E, Majchrzak N, Daniel V, Ferguson Z, Brown S, et al (2017) Spermatogenesis associated 22 is required for DNA repair and synapsis of homologous chromosomes in mouse germ cells. Andrology 5: 299–312. 10.1111/andr.12315 28297563 PMC5354093

[35] Ishishita S, Matsuda Y, Kitada K. (2014) Genetic evidence suggests that Spata22 is required for the maintenance of Rad51 foci in mammalian meiosis. Sci Rep 4: 6148 10.1038/srep06148 25142975 PMC4139951

[36] Dai J, Voloshin O, Potapova S, Camerini-Otero RD. (2017) Meiotic knockdown and complementation reveals essential role of RAD51 in mouse spermatogenesis. Cell Rep 18: 1383–1394. 10.1016/j.celrep.2017.01.024 28178517 PMC5358547

[37] Bradley A, Anastassiadis K, Ayadi A, Battey JF, Bell C, et al (2012) The mammalian gene function resource: The international knockout mouse consortium. Mamm Genome 23: 580–586. 10.1007/s00335-012-9422-2 22968824 PMC3463800

[38] Raymond CS, Soriano P. (2010) ROSA26Flpo deleter mice promote efficient inversion of conditional gene traps in vivo. Genesis 48: 603–606. 10.1002/dvg.20659 20665730 PMC2958239

[39] Abe H, Alavattam KG, Kato Y, Castrillon DH, Pang Q, et al (2018) CHEK1 coordinates DNA damage signaling and meiotic progression in the male germline of mice. Hum Mol Genet 27: 1136–1149. 10.1093/hmg/ddy022 29360988 PMC6185175

[40] Peters AH, Plug AW, van Vugt MJ, de Boer P. (1997) A drying-down technique for the spreading of mammalian meiocytes from the male and female germline. Chromosome Res 5: 66–68. 9088645 10.1023/a:1018445520117

[41] Luo M, Zhou J, Leu NA, Abreu CM, Wang J, et al (2015) Polycomb protein SCML2 associates with USP7 and counteracts histone H2A ubiquitination in the XY chromatin during male meiosis. PLoS Genet 11: e1004954 10.1371/journal.pgen.1004954 25634095 PMC4310598

[42] Reddi PP, Naaby-Hansen S, Aguolnik I, Tsai JY, Silver LM, et al (1995) Complementary deoxyribonucleic acid cloning and characterization of mSP-10: The mouse homologue of human acrosomal protein SP-10. Biol Reprod 53: 873–881. 8547483 10.1095/biolreprod53.4.873

[43] Yang F, De La Fuente R, Leu NA, Baumann C, McLaughlin KJ, et al (2006) Mouse SYCP2 is required for synaptonemal complex assembly and chromosomal synapsis during male meiosis. J Cell Biol 173: 497–507. 10.1083/jcb.200603063 16717126 PMC2063860

[44] Chuma S, Nakatsuji N. (2001) Autonomous transition into meiosis of mouse fetal germ cells in vitro and its inhibition by gp130-mediated signaling. Dev Biol 229: 468–479. 11203703 10.1006/dbio.2000.9989

