# Supplementary figures and images for: Dual functions for the ssDNA-binding protein RPA in meiotic recombination
Source: PLoS Genet. 2019 Feb 4;15(2):e1007952. doi: 10.1371/journal.pgen.1007952 (PMC6375638; doi:10.1371/journal.pgen.1007952)

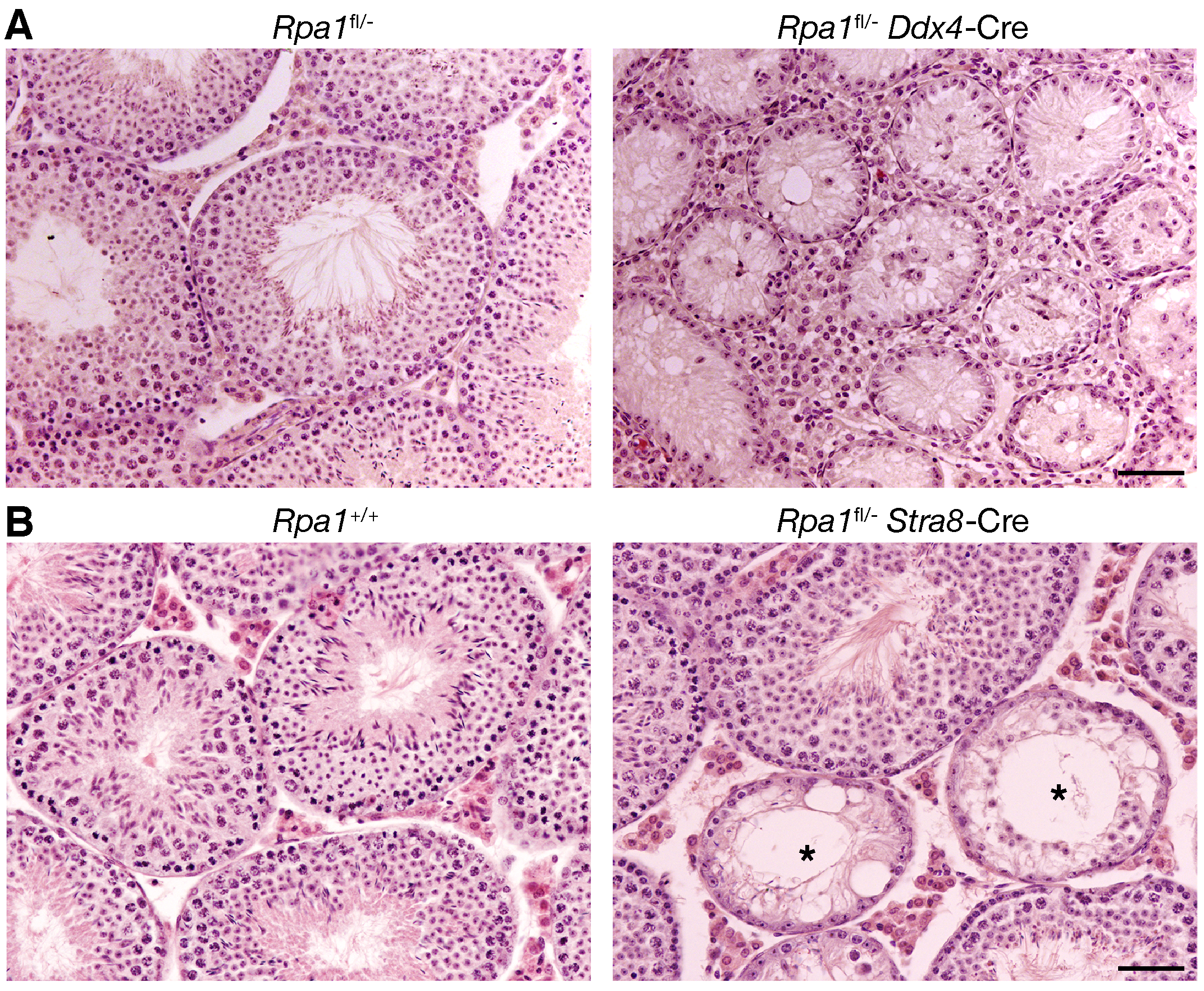

Supplement: S1 Fig — (A) Complete loss of germ cells in testes from 10-week-old Rpa1fl/- Ddx4-Cre males. All seminiferous tubules in the Rpa1cKO males are Sertoli cell only. Rpa1fl/- males are controls. (B) Heterogeneity of testicular histology in 8-week-old Rpa1f/- Stra8-Cre males. Like the wild type control, some seminiferous tubules in the Rpa1 mutant testis have a full spectrum of germ cells, including spermatocytes and spermatids, possibly due to a lack of or inefficient Stra8-Cre-mediated Rpa1 deletion. Other seminiferous tubules in the Rpa1 mutant testis are nearly devoid of all germ cells (marked by asterisks), possibly due to the expression of Stra8-Cre in spermatogonia. Scale bars, 50 μm. (TIF) [file pgen.1007952.s001.tif]

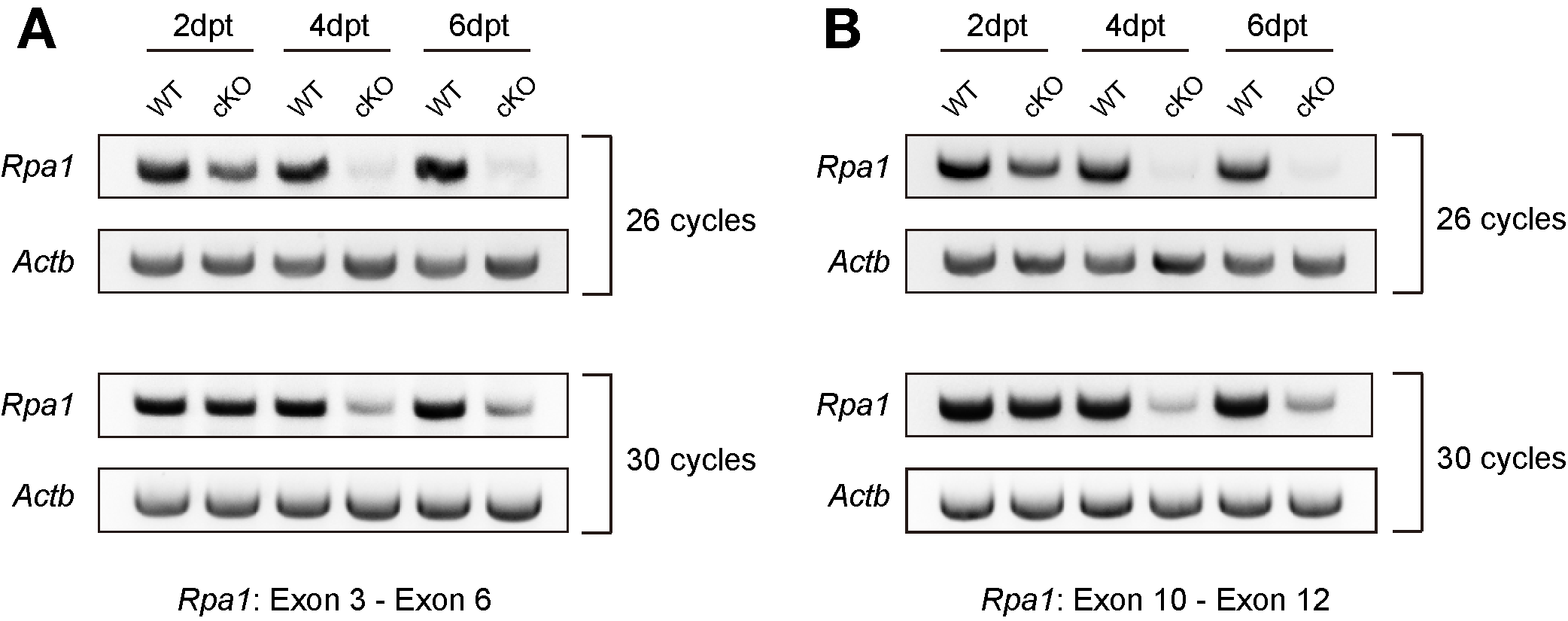

Supplement: S2 Fig — Exon 8 in Rpa1fl allele is flanked by loxP sites (Fig 2A). Deletion of exon 8 results in a frame shift in the resulting Rpa1 mutant transcript. Two RT-PCR assays were designed to amplify the pre-exon 8 region (A) and the post-exon 8 region (B) of Rpa1 transcript. Actb serves a control. dpt, days post-tamoxifen treatment. (TIF) [file pgen.1007952.s002.tif]

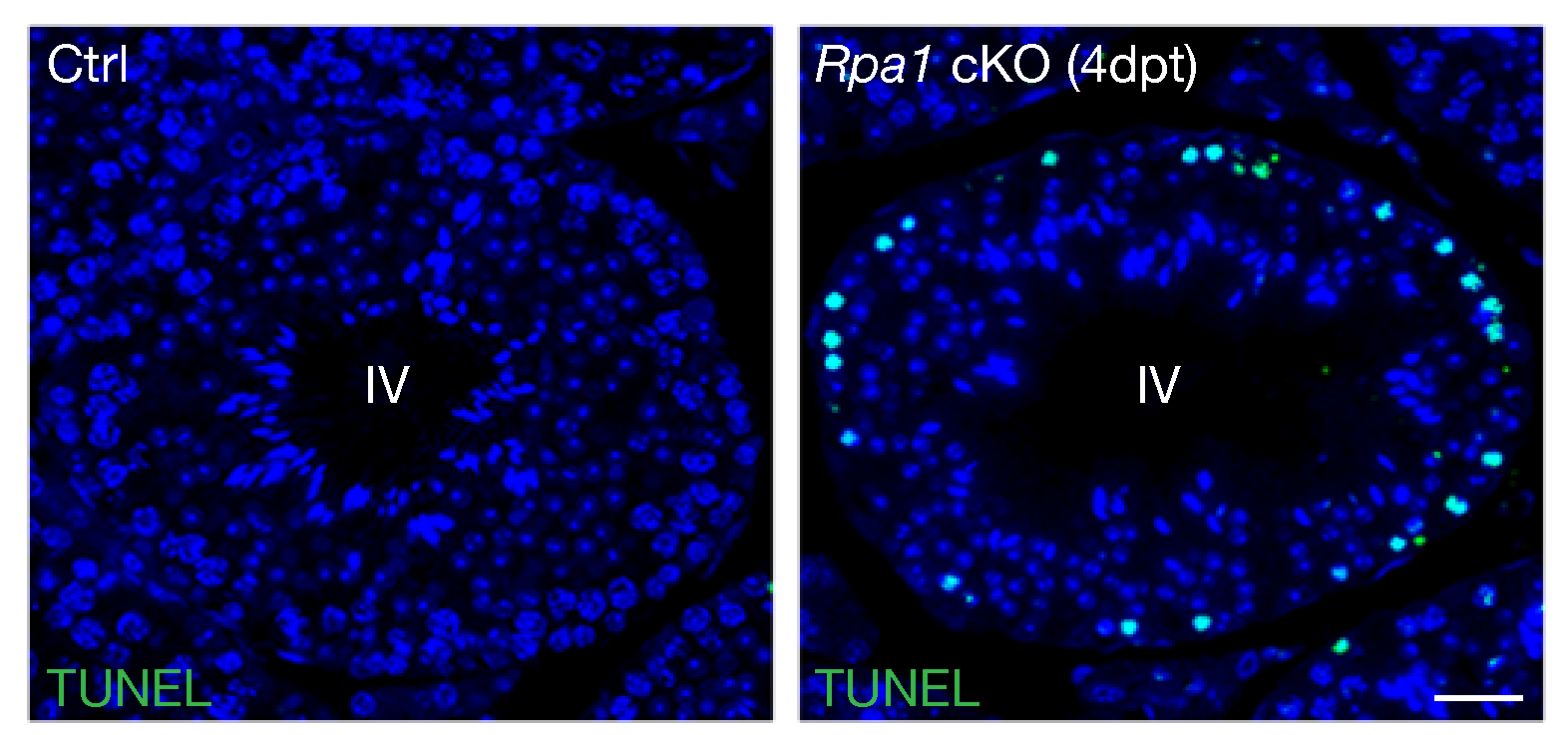

Supplement: S3 Fig — TUNEL analysis was performed on frozen testicular sections from adult control and Rpa1cKO mice at 4 days post-tamoxifen treatment. The seminiferous tubule stage is shown in the middle of the panels. Apoptotic cells are shown in green. DNA was stained with DAPI. Dramatically increased apoptosis in spermatocytes occurs specifically in stage IV Rpa1cKO seminiferous tubules, which correspond to the pachytene checkpoint during male meiosis. Scale bar, 25 μm. (TIF) [file pgen.1007952.s003.tif]

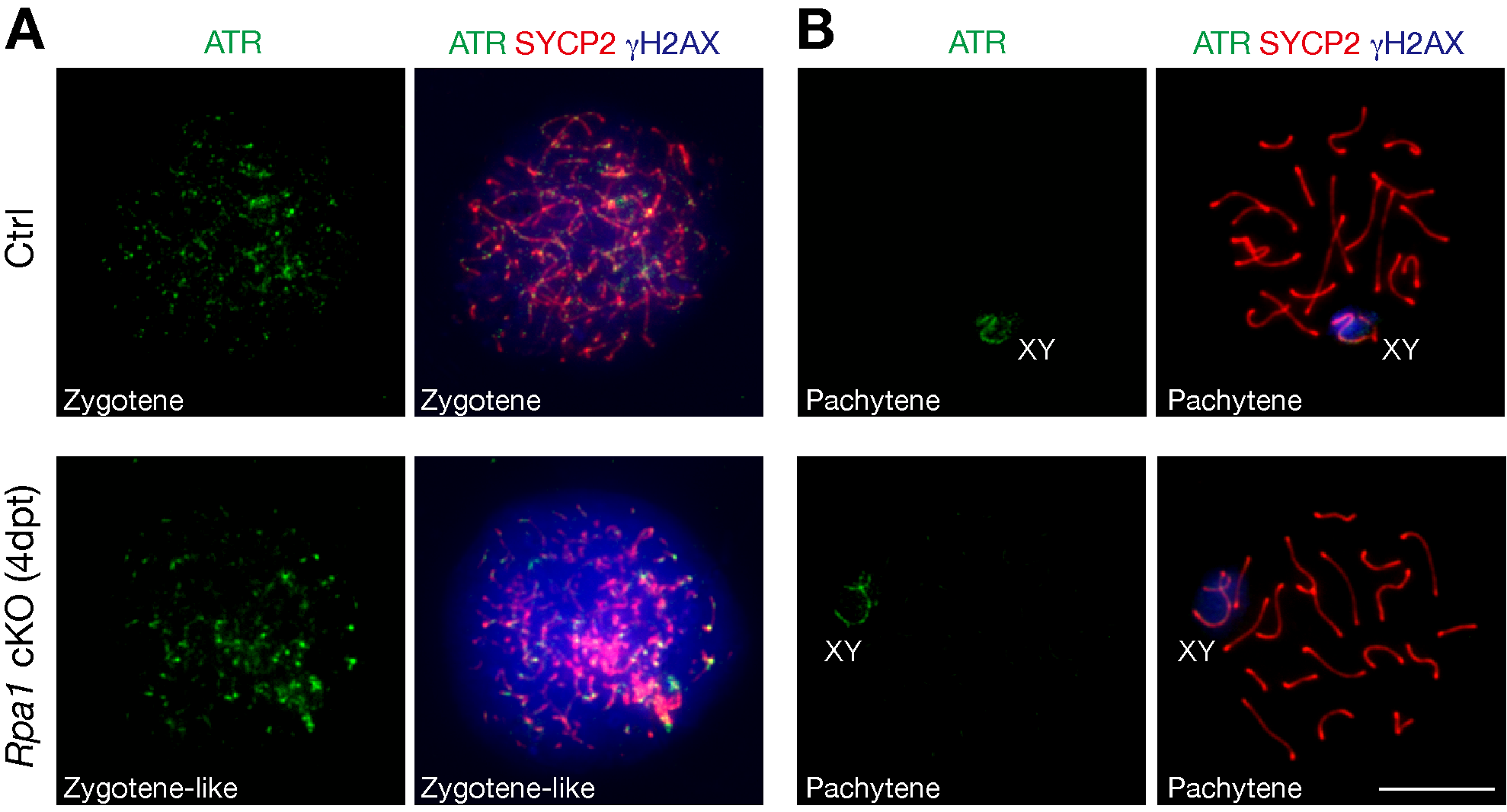

Supplement: S4 Fig — Immunolocalization of ATR was performed in spermatocytes from control and Rpa1cKO testes (4 days post-tamoxifen treatment) at the zygotene and zygotene-like stages (A) and the pachytene stage (B). (TIF) [file pgen.1007952.s004.tif]

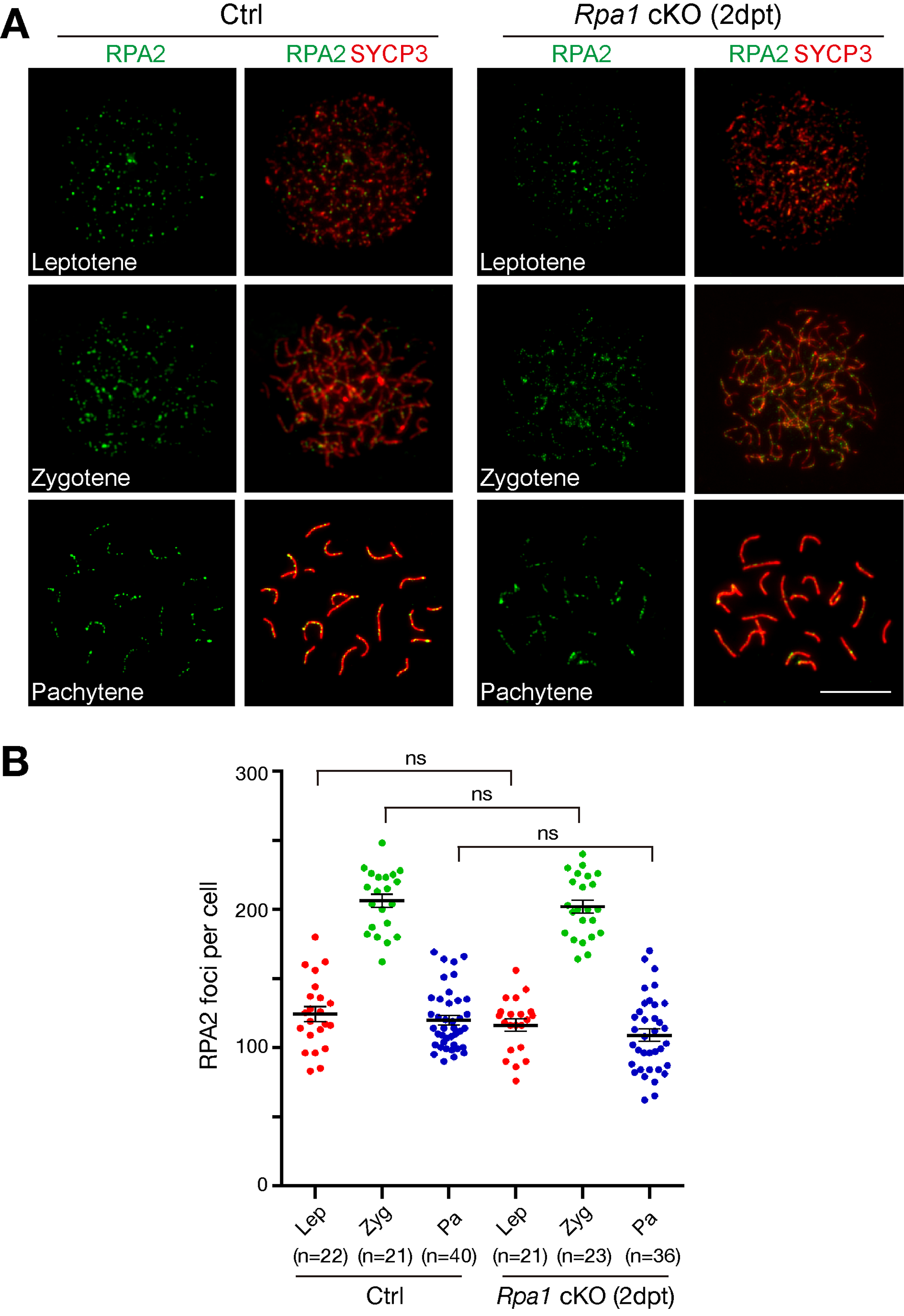

Supplement: S5 Fig — Among three RPA subunits, immunofluorescence of RPA2 on surface spread of spermatocyte nuclei was the strongest. Therefore, RPA2 immunostaining was performed and RPA foci were counted. (A) Presence of RPA2 foci in leptotene, zygotene, and early-mid pachytene spermatocytes from both control and Rpa1cKO mice at 2 dpt. Scale bar, 25 μm. (B) Quantification of RPA2 foci in control and Rpa1cKO spermatocytes. n, number of spermatocytes; ns, not statistically significant. (TIF) [file pgen.1007952.s005.tif]

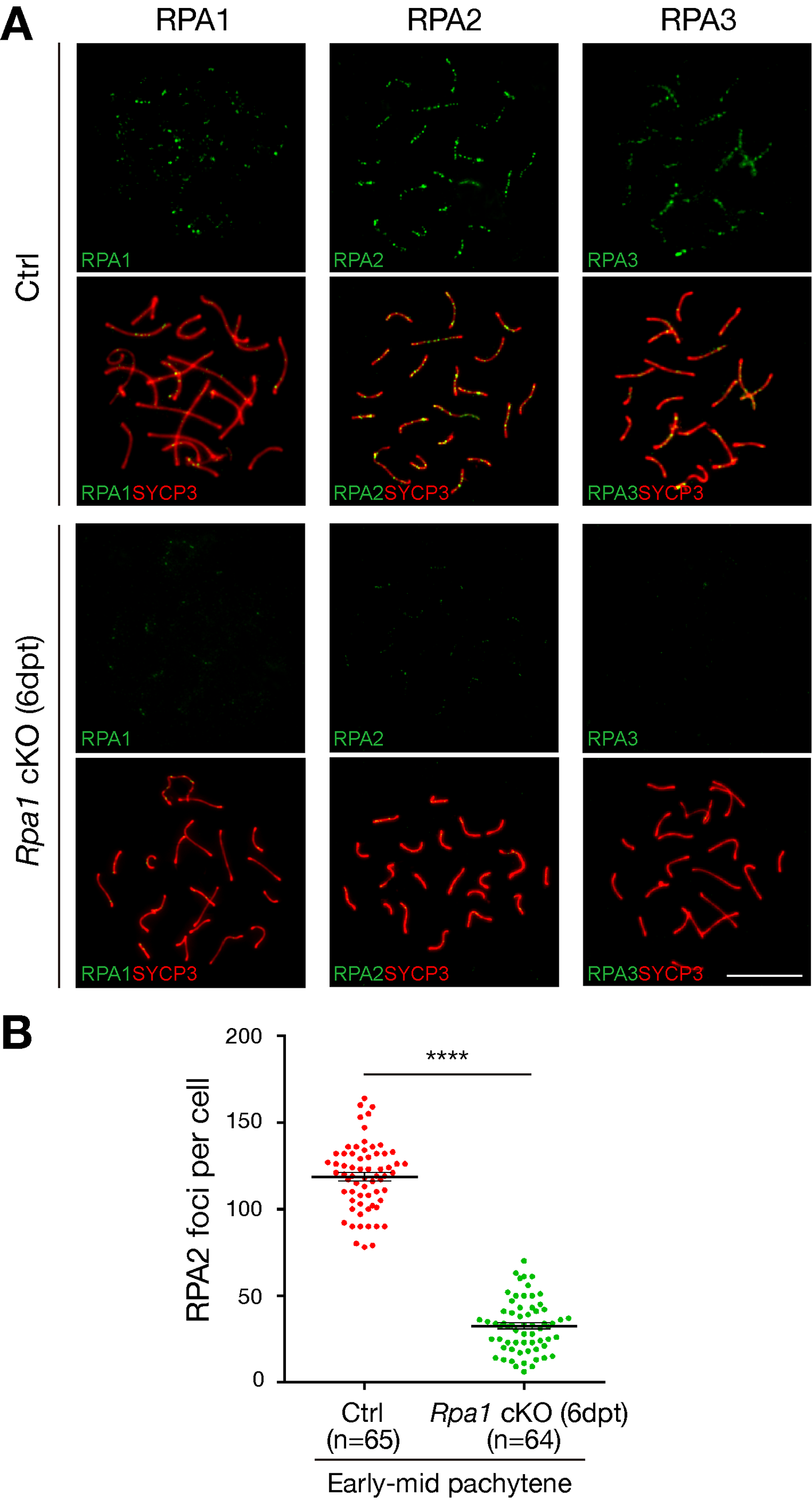

Supplement: S6 Fig — (A) RPA1, RPA2, and RPA3 form foci on the synaptonemal complex in control early-mid pachytene spermatocytes (top panels) but the RPA foci are sharply reduced in number and intensity in Rpa1cKO early-mid pachytene spermatocytes (bottom panels). Scale bar, 25 μm. (B) Quantification of RPA2 foci in control and Rpa1cKO (6 dpt) early-mid pachytene spermatocytes. n, number of early-mid pachytene spermatocytes. (TIF) [file pgen.1007952.s006.tif]

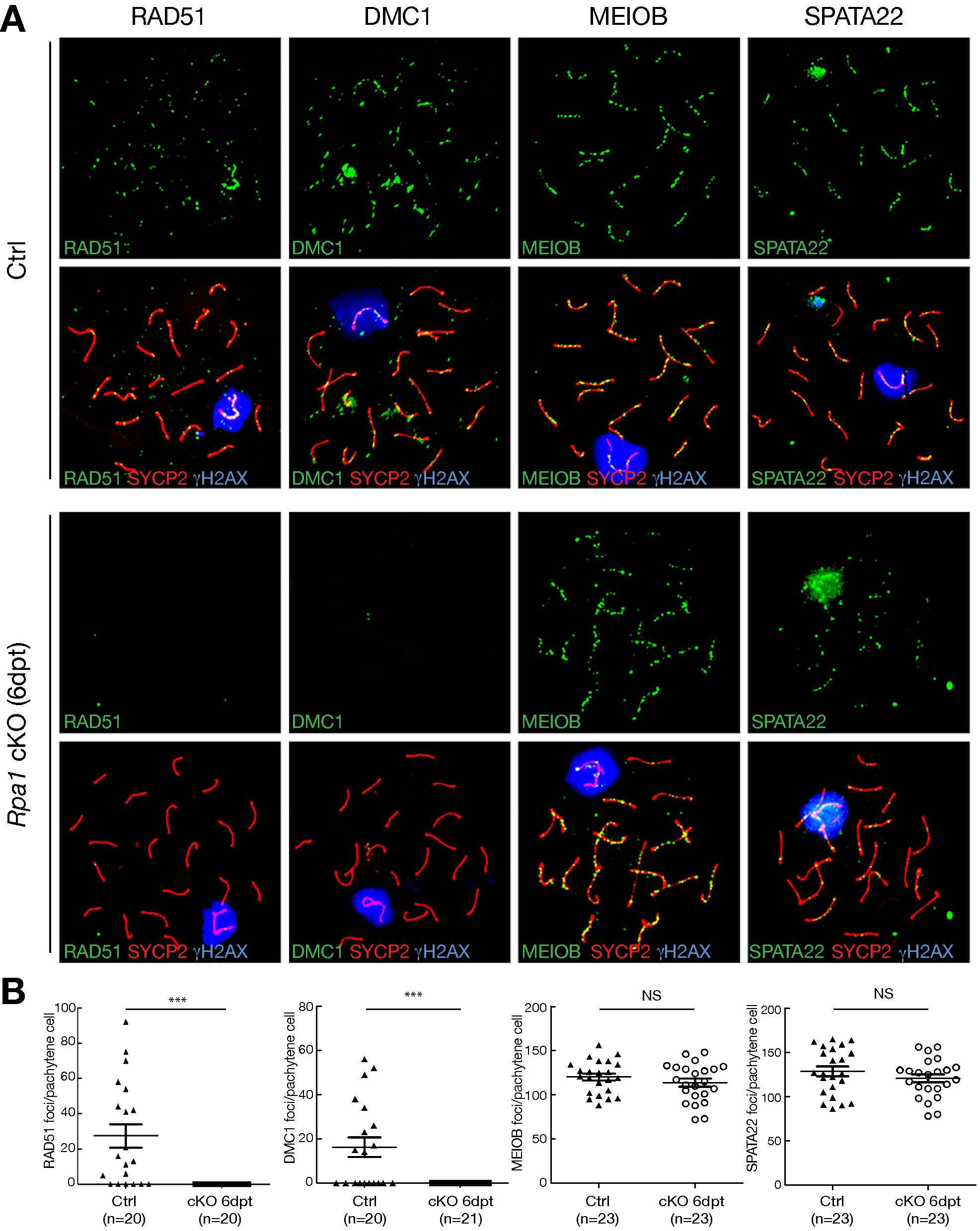

Supplement: S7 Fig — (A) Immunolocalization of ssDNA-binding proteins in control and Rpa1cKO early/mid- pachytene (6 days post-tamoxifen treatment) spermatocytes. Synaptonemal complexes were immunostained with anti-SYCP2 antibody. Only XY body is γH2AX-positive. (B) Dot plots of foci of RAD51, DMC1, MEIOB, and SPATA22 in control and Rpa1cKO early/mid pachytene spermatocytes. Solid lines show the average ± SD. n, number of cells counted from two experiments; ***, p < 0.001 (Student’s t test); ns, non-significant. (TIF) [file pgen.1007952.s007.tif]
