# Supplementary material for: Dual functions for the ssDNA-binding protein RPA in meiotic recombination
Source: PLoS Genet. 2019 Feb 4;15(2):e1007952. doi: 10.1371/journal.pgen.1007952 (PMC6375638; doi:10.1371/journal.pgen.1007952)
Supplement: S1 Table — (DOCX) [file pgen.1007952.s008.docx]

**S1 Table. Primary antibodies used in this study.**

| Antibody | Host | Producer | Cat. No/reference | Dilution | |
| --- | --- | --- | --- | --- | --- |
|  |  |  |  | WB | IF |
| RPA1 | Rabbit | Abcam | Ab87272 | 1:1000 | 1:50 |
| RPA2 | Rabbit | Custom-made | UP2436 (this study) | 1:250 | 1:50 |
| RPA2 | Guinea pig | Custom-made | GP111 (this study) |  | 1:50 |
| RPA3 | Rabbit | Custom-made | UP2439 (this study) | 1:250 | 1:50 |
| MEIOB | Rabbit | Custom-made | [13] | 1:2000 | 1:50 |
| MEIOB | Guinea pig | Custom-made | [13] |  | 1:50 |
| SPATA22 | Rabbit | ProteinTech Group | 16989-1-AP | 1:200 | 1:50 |
| DMC1 | Rabbit | ProteinTech Group | 13714-1-AP |  | 1:30 |
| DMC1 | Rabbit | Santa Cruz | Sc-22768 H-100 |  | 1:30 |
| DMC1 | Goat | Santa Cruz | Sc-8973 C-20 | 1:1000 |  |
| RAD51 | Rabbit | Santa Cruz | Sc-8349 H-92 | 1:250 |  |
| RAD51 | Rabbit | Millipore | PC-130 |  | 1:25 |
| ATR | Rabbit | Cell Signalling | 2790 |  | 1:50 |
| γH2AX | Mouse | Millipore | 05-636 |  | 1:200 |
| TEX11 | Rabbit | Custom-made | [29] |  | 1:50 |
| MSH4 | Rabbit | Abcam | Ab58666 |  | 1:50 |
| SYCP1 | Rabbit | Abcam | Ab15090 |  | 1:200 |
| SYCP2 | Guinea pig | Custom-made | UPGP21 [43] |  | 1:200 |
| SYCP3 | Mouse | Abcam | Ab97672 |  | 1:100 |
| SYCP3 | Rabbit | A gift from S. Chuma | [44] |  | 1:100 |
| BrdU | Rat | Abcam | Ab6326 |  | 1:50 |
| SP10 | Guinea pig | A gift from P.P.Reddi | [42] |  | 1:250 |
| ACTB | Mouse | Sigma | A5441 | 1:5000 |  |
